# Crystal structure and absolute configuration of (3*S*,4a*S*,8a*S*)-*N*-*tert*-butyl-2-[(*S*)-3-(2-chloro-4-nitro­benzamido)-2-hy­droxy­prop­yl]deca­hydro­isoquinoline-3-carboxamide and (3*S*,4a*S*,8a*S*)-*N*-*tert*-butyl-2-{(*S*)-2-[(*S*)-1-(2-chloro-4-nitro­benzoyl)pyrrolidin-2-yl]-2-hy­droxy­eth­yl}deca­hydro­iso­quinoline-3-carboxamide

**DOI:** 10.1107/S2056989015020046

**Published:** 2015-10-31

**Authors:** Tucker Maxson, Jeffery A. Bertke, Danielle L. Gray, Douglas A. Mitchell

**Affiliations:** a Department of Chemistry, University of Illinois, 345 Roger Adams Lab, 600 South Mathews Avenue, Urbana, IL 61801, USA; b University of Illinois, School of Chemical Sciences, Box 59-1, 505 South Mathews Avenue, Urbana, Illinois 61801, USA

**Keywords:** crystal structure, chiral crystal, absolute configuration, nelfinavir, HIV protease inhibitor

## Abstract

The crystal structure and absolute configuration of the two new title nelfinavir analogs, C_24_H_35_ClN_4_O_5_, (I), and C_27_H_39_ClN_4_O_5_, (II), have been determined. Each of these mol­ecules exhibits a number of disordered moieties. There are intra­molecular N—H⋯O hydrogen bonds in both (I) and (II). In (I) it involves the two carboxamide groups, while in (II) it involves the *N*-*tert*-butyl carboxamide group and the 2-hydroxyl O atom. The inter­molecular hydrogen bonding in (I) (O—H⋯O and N—H⋯O) leads to two-dimensional sheets that extend parallel to the *ac* plane. The inter­molecular hydrogen bonding in (II) (O—H⋯O) leads to chains that extend parallel to the *a* axis.

## Abstract 2

The crystal structure and absolute configuration of two new nelfinavir analogs have been determined. Inter­molecular hydrogen bonding leads to two-dimensional sheets in one analog and one-dimensional chains in the other.

## Chemical context   

Nelfinavir (Viracept) is an FDA approved HIV protease inhibitor identified through structure-based design with a low nanomolar inhibitory concentration against the HIV aspartyl protease (Kaldor *et al.*, 1997 ▸). Although nelfinavir is no longer recommended as a first-line treatment against HIV due to its inferior efficacy compared to alternative protease inhibitors (Panel on Anti­retroviral Guidelines, 2015 ▸), it has been found to have a number of additional biological activities that may have therapeutic utility, including anti­viral (against other human viruses) (Yamamoto *et al.*, 2004 ▸; Kalu *et al.*, 2014 ▸), anti­cancer (Gantt *et al.*, 2013 ▸; Koltai, 2015 ▸), and anti­virulence activity (Maxson *et al.*, 2015 ▸). However, nelfinavir was originally designed with only the HIV protease active site in mind and the structure is likely not optimal for binding to the alternative targets involved in these other activities. We recently reported on the synthesis of a collection of nelfinavir analogs that may be of inter­est for efforts to repurpose the drug (Maxson *et al.*, 2015 ▸).

**Figure 1:**
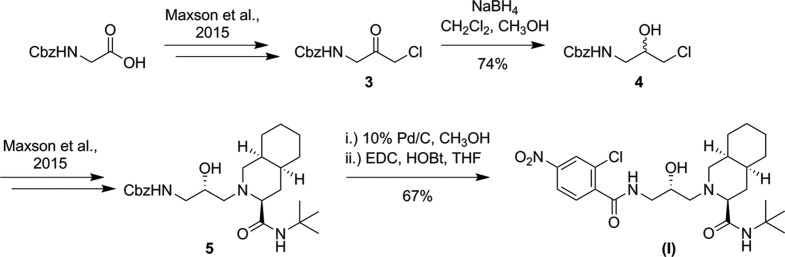
The synthesis of (I).

**Figure 2:**
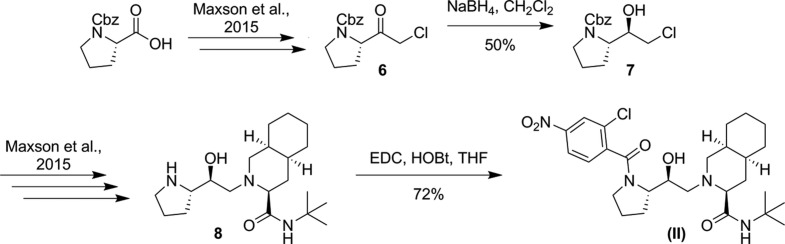
The synthesis of (II).

The syntheses of the title compounds were achieved by a previously reported route that utilizes the configuration of the amino acid starting material to control the stereochemical outcome of the sodium borohydride reduction of the chloro­methyl ketone (Kaldor *et al.*, 1997 ▸). However, the reduction of compound (I), derived from achiral glycine, results in a racemic mixture (Fig. 1 ▸), while the reduction of compound (II), derived from l-proline, does not benefit from a strong directing influence from the existing chiral center (Fig. 2 ▸). The products of the two reductions were carried forward through the remainder of each synthesis to generate the title compounds. The absolute configurations of compounds (I) and (II), as well as the conformations they adopt due to the increased flexibility and rigidity, respectively, relative to nelfinavir was investigated by X-ray diffraction.
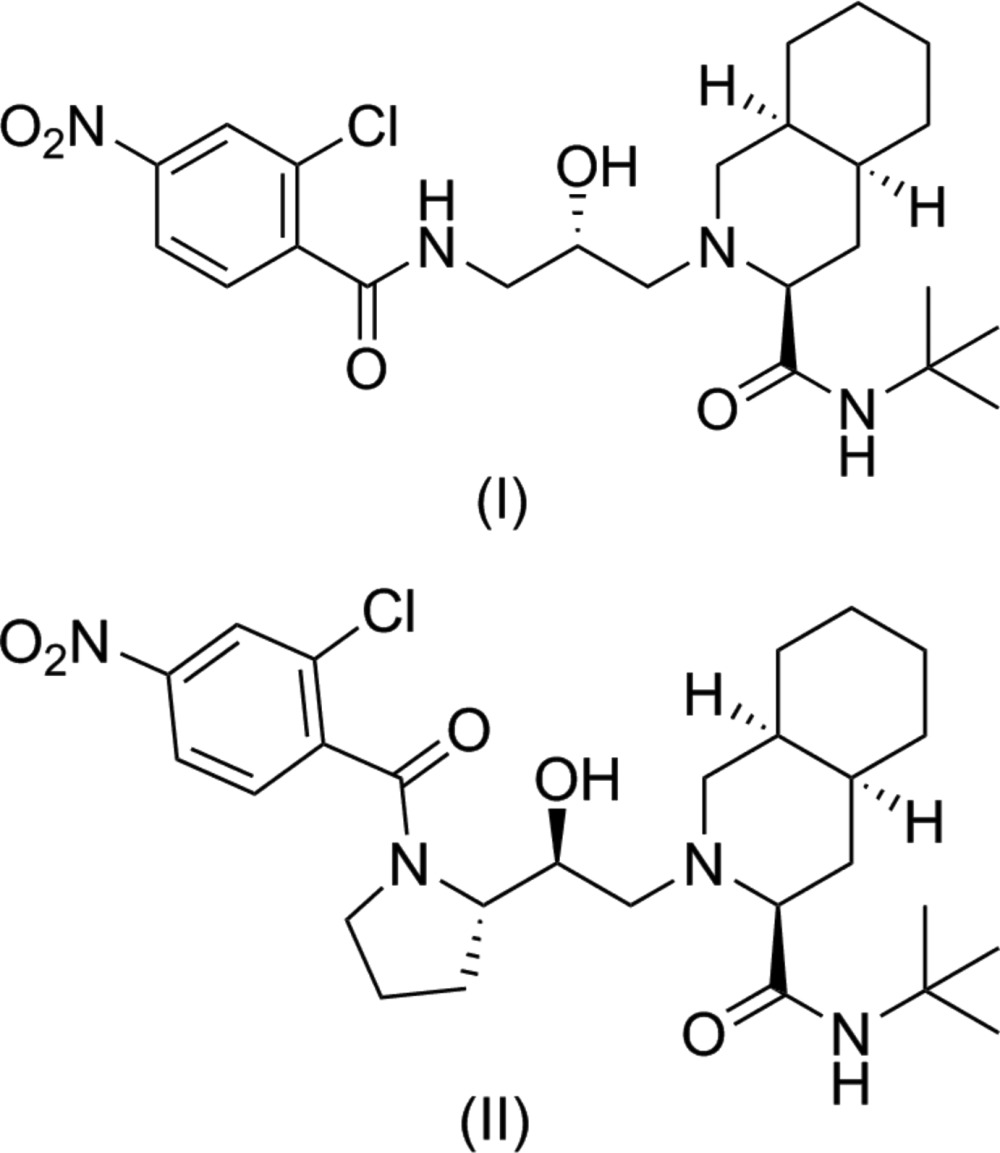


## Structural commentary   

**Figure 3:**
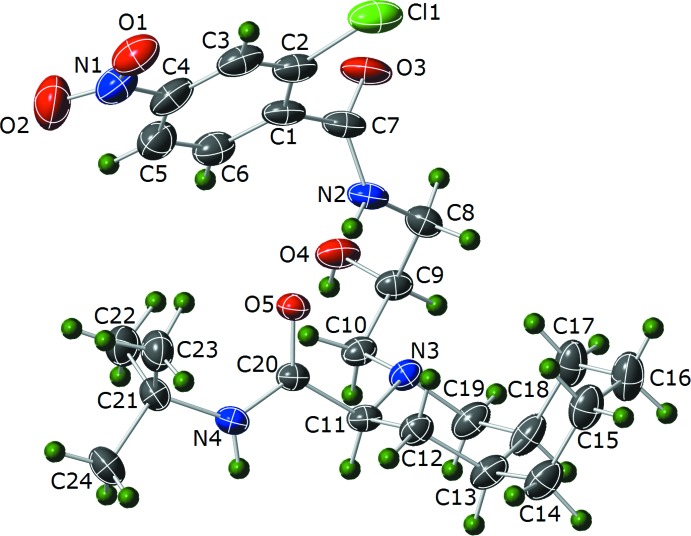
Plot showing 35% probability ellipsoids for non-H atoms and circles of arbitrary size for H atoms for (I). Only the major component of disordered sites is shown.

**Figure 4:**
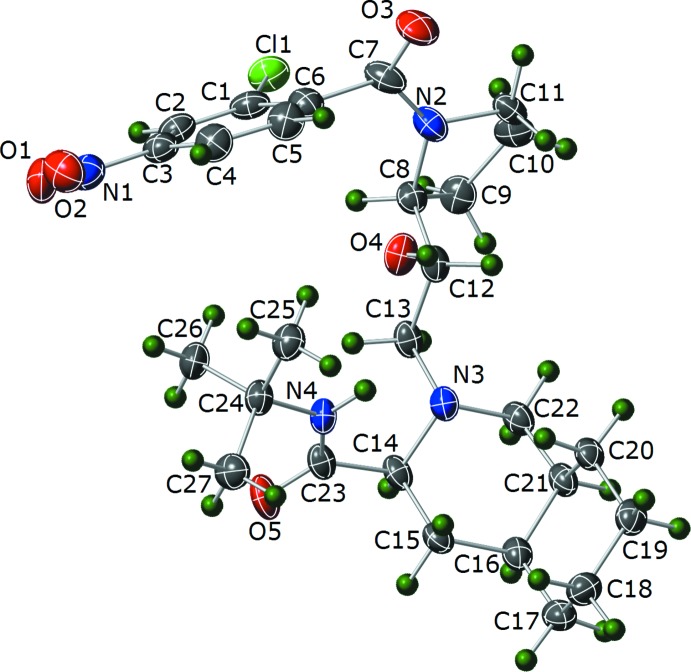
Plot showing 35% probability ellipsoids for non-H atoms and circles of arbitrary size for H atoms for (II). Only the major component of disordered sites is shown.

The core mol­ecular structures of (I) and (II) are comprised of *N*-*tert*-butyl-2-(2-hy­droxy­alk­yl)deca­hydro­iso­quinoline-3-carb­ox­amide groups. The difference between the two species comes from the substitution at the N position of the deca­hydro­iso­quinoline group. Compound (I) has a (2-chloro-4-nitro­benzamido)-2-hy­droxy­propyl group at the N-atom position of the deca­hydro­iso­quinoline ring (Fig. 3 ▸). Compound (II) has a (2-chloro-4-nitro­benzo­yl)pyrrolidin-2-yl)-2-hy­droxy­ethyl group at the N-atom position (Fig. 4 ▸).

There is disorder of the Cl group in (I) over two positions with the site occupancies refining to 0.941 (8) and 0.059 (8) for Cl1 and Cl1*B*, respectively. The nitro group is disordered over two positions, with the site occupancies refining to 0.60 (2) and 0.40 (2). The NO_2_ group in one orientation is essentially coplanar with the phenyl ring [O1*B*—N1*B*—C4—C3; τ = 1(2)°] and in the other orientation is twisted slightly more out of plane [O1—N1—C4—C3; τ = −9.0 (13)°]. Both six-membered rings of the deca­hydro­iso­quinoline group in (I) adopt a chair conformation, the dihedral angle between the best-fit planes of the cyclo­hexyl and piperidine moieties is 119.9 (15)°. There is one intra­molecular hydrogen-bonding inter­action in (I) which involves the two carboxamide groups (N2—H2⋯O5; Table 1 ▸). The Flack *x* parameter of −0.008 (18) and the Hooft *y* parameter of −0.010 (19) indicate that the absolute configuration of (I) has been assigned correctly.

There are multiple disordered moieties in (II), the nitro group is disordered over two positions with the site occupancies for the two orientations refining to 0.967 (6) and 0.033 (8). In both orientations, the NO_2_ group is twisted out of the plane of the phenyl ring; the major orientation is twisted out of the plane less [O1—N1—C3—C2; τ = 10.9 (4)°] than the minor orientation [O1*B*—N1*B*—C3—C2; τ = −26 (6)°]. The carbonyl C7—O3 group is disordered over two positions, with the site occupancies refining to 0.58 (2) and 0.42 (2). In the minor orientation, the CO group is nearly normal to the plane of the phenyl ring [O3*B*—C7*B*—C6—C5; τ = −89 (3)°], while the major orientation is significantly less out of plane [O3—C7—C6—C5; τ = −44 (3)°]. The final two disordered moieties of (II) are a portion of the pyrrolidin-2-yl group and the three methyl groups of *tert*-butyl. The C10 and C11 atoms of the pyrrolidin-2-yl group are disordered over two positions, with site occupancies of 0.669 (16) and 0.331 (16). The *tert*-butyl methyl groups are also disordered over two positions *via* a slight rotation around the N4—C24 bond, the site occupancies refining to 0.811 (17) and 0.189 (17). Similar to (I), both six-membered rings of the deca­hydro­iso­quinoline group in (II) adopt a chair conformation, with a dihedral angle between the best-fit planes of the cyclo­hexyl and piperidine moieties of 116.3 (17)°. There is one weak intra­molecular hydrogen-bonding inter­action in (II), involving the *N*-*tert*-butyl carboxamide group and the 2-hydroxyl O atom (N4—H4*C*⋯O4; Table 2 ▸). The Flack *x* parameter of 0.036 (19) and the Hooft *y* parameter of 0.03 (2) indicate that the absolute configuration of (II) has been assigned correctly.

## Supra­molecular features   

**Figure 5:**
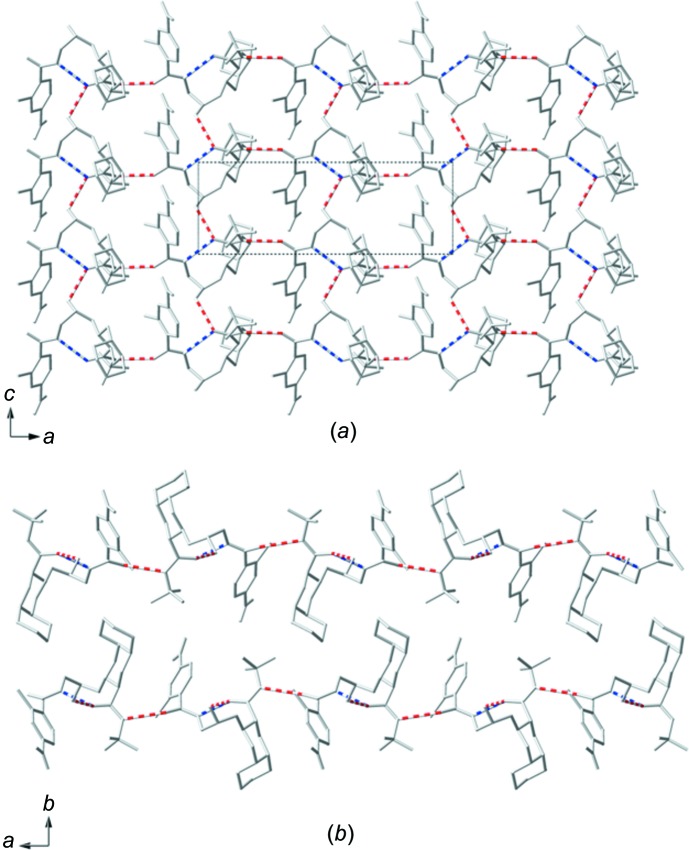
A plot of the packing of (I) viewed (*a*) along the *b* axis, showing a hydrogen-bonded two-dimensional sheet overlaid with the unit cell, and (*b*) along the *c* axis, showing how two layers stack together along the *b* axis. Only the major component of disordered sites are shown. Red dashed lines indicate inter­molecular hydrogen bonding and blue dashed lines indicate intra­molecular hydrogen bonding.

The extended structure of (I) is a two-dimensional sheet of hydrogen-bonded mol­ecules extending in the *ac* plane (Fig. 5 ▸ *a*). Each mol­ecule of (I) is hydrogen bonded to four neighboring mol­ecules *via* O—H⋯O and N—H⋯O inter­actions; the details of these inter­actions can be found in Table 1 ▸. The two-dimensional layers stack in an *ABAB* pattern along the crystallographic *b* axis (Fig. 5 ▸ *b*). The layers are separated by the bulky deca­hydro­iso­quinoline groups, which protrude above and below the sheets. The layers alternate between these bulky groups pointing ‘left’ and ‘right’, this along with a slight offset between the *A* and *B* layers allows them to inter­digitate.

**Figure 6:**
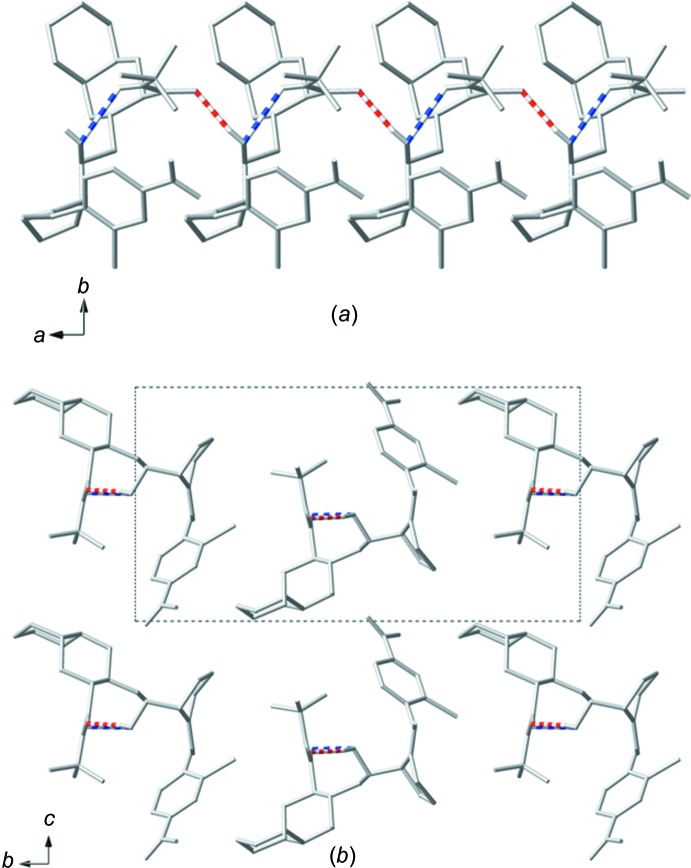
A plot of the packing of (II) viewed (*a*) along the *c* axis, showing a hydrogen-bonded one-dimensional chain, and (*b*) along the *a* axis, showing how the one-dimensional chains pack together overlaid with the unit cell. Only the major component of disordered sites is shown. Red dashed lines indicate inter­molecular hydrogen bonding and blue dashed lines indicate intra­molecular hydrogen bonding.

The extended structure of (II) is a one-dimensional chain of hydrogen-bonded mol­ecules extending parallel to the crystallographic *a* axis (Fig. 6 ▸ *a*). Each mol­ecule of (II) is hydrogen bonded to two neighboring mol­ecules *via* O—H⋯O inter­actions, the details of these inter­actions can be found in Table 2 ▸. The one-dimensional chains are separated by the bulky deca­hydro­iso­quinoline groups and the *tert*-butyl groups, which prevent the chains from linking *via* further hydrogen-bonding inter­actions (Fig. 6 ▸ *b*).

## Database survey   

A search of the Cambridge Crystallographic Database (CSD; Groom & Allen, 2014 ▸) returns only three crystal structures with the *N*-(*tert*-but­yl)deca­hydro­iso­quinoline-3-carboxamide core. One of the structures is *N*-(*tert*-but­yl)deca­hydro­iso­quinoline-3-carboxamide (CSD refcode COVYAO; Zhao *et al.*, 2006 ▸). The other two mol­ecules are nelfinavir derivatives like (I) and (II), which were isolated during optimization of the synthesis. The difference between these two mol­ecules comes *via* the substitution at the N-atom position of the deca­hydro­iso­quinoline group.

One compound has a 3-amino-2-hy­droxy-4-(phenyl­sulfan­yl)butyl group in this position (CSD refcode QONJUY; Inaba *et al.*, 2000 ▸) and the other has a 3-acet­oxy-2-(3-acet­oxy-2-methyl­benzoyl­amino)-4-(phenyl­sulfan­yl)butyl group at the N-atom position (CSD refcode GONKOJ; Inaba *et al.*, 1998 ▸). Each of these mol­ecules has intra­molecular N—H⋯O hydrogen bonding. In QONJUY it involves the two carboxyamide groups similar to the situation in compound (I). In GONKOJ it involves the *N*-*tert*-butyl carboxamide group and the 2-hydroxyl O atom similar to the situation in compound (II). The core structure of each of these previously reported materials is similar to (I) and (II) in that both six-membered rings of the deca­hydro­iso­quinoline groups adopt chair conformations. The dihedral angle between the best-fit planes of the cyclo­hexyl and piperidine moieties for the 3-amino-2-hy­droxy-4-(phenyl­sulfan­yl)butyl-substituted mol­ecule is 117.1 (18)°. Similarly, this angle for the 3-acet­oxy-2-(3-acet­oxy-2-methyl­benzoyl­amino)-4-(phenyl­sulfan­yl)butyl-substituted mol­ecule is 116.8 (14)°.

## Synthesis and crystallization   

Compound (I) was synthesized through the inter­mediate chloro­methyl hy­droxy **4** (Fig. 1 ▸). Chloro­methyl ketone **3** (571 mg, 2.36 mmol) was dissolved in di­chloro­methane (7 ml) and methanol (4 ml) under nitro­gen. The reaction was cooled to 273 K and sodium borohydride (63 mg, 1.65 mmol) was added in one portion. The reaction was stirred cold for 1h before being quenched by the slow addition of 2 *M* HCl (2 ml). The reaction was dried and the solid was dissolved in ethyl acetate. The product was washed twice with water and once with brine, dried over sodium sulfate, and concentrated by rotary evaporation. The product was purified by silica flash column chromatography (gradient of 0–8% EtOAc in DCM) to yield racemic **4** as a colorless oil (yield 423 mg, 75% yield). ^1^H NMR (500 MHz, CDCl_3_): δ 7.33–7.28 (complex, 5H), 5.63 (*t*, *J* = 6 Hz, 1H), 5.06 (*s*, 2H), 3.88 (*s*, 2H), 3.48 (*m*, 2H), 3.39 (*m*, 1H), 3.22 (*m*, 1H). ^13^C NMR (500 MHz, CDCl_3_): δ 157.23, 135.93, 128.36, 128.06, 127.91, 70.52, 66.90, 46.44, 43.96. HRMS (*m*/*z*): [*M* + H]^+^ calculated for C_11_H_15_ClNO_3_, 244.0740; observed, 244.0741. For the synthesis of compound (I), compound **5** (104 mg, 0.233 mmol) was dissolved in methanol (15 ml) with 10% palladium on carbon (74 mg, 0.070 mmol). The solution was degassed for 30 min before being placed under 1 atm of hydrogen and stirred for 2 h at room temperature. The reaction was filtered through celite, dried to a solid, and taken up in tetra­hydro­furan (5 ml). 2-Chloro-4-nitro­benzoic acid (52 mg, 0.256 mmol), 3-[3-(di­methyl­amino)­prop­yl]-1-ethyl­carbodi­imide hydro­chloride (49 mg, 0.256 mmol), and hy­droxy­benzotriazole hydrate (42 mg, 0.256 mmol) were added and the reaction was stirred at room temperature overnight. The reaction was taken up in ethyl acetate, washed once with sodium bicarbonate and once with brine, and dried over sodium sulfate. The product was purified by silica flash-column chromatography (gradient of 0–3% MeOH in DCM) to yield (I) as a yellow solid (yield 77 mg, 67%). Crystals suitable for X-ray diffraction were obtained from the vapor diffusion of pentane into a solution of compound (I) in ethyl acetate at room temperature. ^1^H NMR (500 MHz, CDCl_3_): δ 8.41 (*q*, *J* = 4 Hz, 1H), 8.24 (*d*, *J* = 2 Hz, 1H), 8.13 (*dd*, *J*1 = 2 Hz, *J*2 = 8.5 Hz, 1H), 7.76 (*d*, *J* = 8.5 Hz, 1H), 5.60 (*s*, 1H), 4.04 (*m*, 2H), 3.47 (*dt*, *J*1 = 4 Hz, *J*2 = 14 Hz, 1H), 3.35 (*br*, 1H), 2.71 (*dd*, *J*1 = 2 Hz, *J*2 = 11.5 Hz, 1H), 2.49 (*dd*, *J*1 = 3 Hz, *J*2 = 11.5 Hz, 1H), 2.36 (*dd*, *J*1 = 10 Hz, *J*2 = 12.5 Hz, 1H), 2.22 (*dd*, *J*1 = 5 Hz, *J*2 = 12.5 Hz, 1H), 2.18 (*dd*, *J*1 = 3 Hz, *J*2 = 11.5 Hz, 1H), 1.95 (*q*, *J* = 12 Hz, 1H), 1.80–1.08 (complex, 20H). ^13^C NMR (500 MHz, CDCl_3_): δ 174.16, 167.06, 148.39, 142.00, 132.80, 130.18, 124.96, 121.56, 70.40, 68.29, 59.09, 57.54, 51.27, 43.27, 35.83, 33.55, 31.02, 30.86, 28.39, 26.19, 25.52, 20.18. HRMS (*m*/*z*): [*M* + H]^+^ calculated for C_24_H_36_ClN_4_O_5_, 495.2374; observed, 495.2376.

Compound (II) was synthesized through the inter­mediate chloro­methyl hydroxyl **7** (Fig. 2 ▸). Chloro­methyl ketone **6** (860 mg, 3.05 mmol) was dissolved in di­chloro­methane (7 ml) and methanol (4 ml) under nitro­gen. The reaction was cooled to 273 K and sodium borohydride (81 mg, 2.14 mmol) was added in one portion. The reaction was stirred cold for 1h before being quenched by the slow addition of 2 *M* HCl (2 ml). The reaction was dried and the solid was dissolved in ethyl acetate. The product was washed twice with water and once with brine, dried over sodium sulfate, and concentrated by rotary evaporation. Thin-layer chromatography (TLC) analysis showed two diastereomers with the higher *R*_F_ compound being the (*S*,*R*) product. Both diastereomers were purified by silica flash-column chromatography (gradient of 0–10% EtOAc in DCM) to yield the (*S*,*S*)-isomer as a white solid (yield 279 mg, 32%) and (*S*,*R*)-isomer (7) as a white solid (yield 429 mg, 50%). Characterization of the (*S*,*R*)-isomer (7): ^1^H NMR (500 MHz, CDCl_3_): δ 7.37–7.28 (complex, 5H), 5.13 (*dd*, *J*1 = 12.5 Hz, *J*2 = 16 Hz, 2H), 4.95 (*d*, *J* = 2 Hz, 1H), 4.11 (*m*, 1H), 3.81 (*br s*, 1H), 3.72 (*d*, *J* = 11 Hz, 1H), 3.55 (*m*, 2H), 3.37 (*m*, 1H), 2.03 (*m*, 1H), 1.89 (*m*, 1H), 1.81 (*m*, 1H), 1.72 (*m*, 1H). ^13^C NMR (500 MHz, CDCl_3_): δ 157.52, 136.04, 128.27, 127.87, 127.65, 74.69, 67.22, 60.57, 47.91, 47.05, 28.12, 23.94. HRMS (*m*/*z*): [*M* + H]^+^ calculated for C_14_H_19_ClNO_3_, 284.1053; observed, 284.1055. For the synthesis of compound (II), compound **8** (218 mg, 0.620 mmol) was dissolved in tetra­hydro­furan (6 ml) with 2-chloro-4-nitro­benzoic acid (138 mg, 0.682 mmol), 3-[3-(di­methyl­amino)­prop­yl]-1-ethyl­carbodi­imide hydro­chloride (131 mg, 0.682 mmol), and hy­droxy­benzotriazole hydrate (111 mg, 0.682 mmol). The reaction was stirred at room temperature overnight. The reaction was taken up in ethyl acetate, washed once with sodium bicarbonate and once with brine, and dried over sodium sulfate. The product was purified by silica flash-column chromatography (gradient of 0–5% MeOH in DCM) to yield (II) as a yellow solid (yield 248 mg, 72%). Crystals suitable for X-ray diffraction were obtained by layering pentane over a solution of compound (II) in di­chloro­methane at room temperature. ^1^H NMR (500 MHz, CDCl_3_): δ 8.31 (*d*, *J* = 2 Hz, 1H), 8.20 (*dd*, *J*1 = 2 Hz, *J*2 = 8.5 Hz, 1H), 7.54 (*d*, *J* = 8.5 Hz, 1H), 6.87 (*s*, 1H), 5.31 (*s*, 1H), 4.36 (*m*, 1H), 3.99 (*m*, 1H), 3.24 (*m*, 2H), 2.91 (*d*, *J* = 11 Hz, 1H), 2.63 (*m*, 2H), 2.18–1.13 (complex, 26H). ^13^C NMR (500 MHz, CDCl_3_): δ 173.83, 172.95, 148.31, 142.45, 128.53, 124.96, 122.35, 121.69, 69.81, 69.73, 60.88, 58.37, 57.98, 50.55, 50.51, 49.05, 35.84, 33.23, 31.07, 30.80, 28.56, 28.20, 26.20, 25.46, 24.53, 20.16. HRMS (*m*/*z*): [*M* + H]^+^ calculated for C_27_H_40_N_4_O_5_Cl, 535.2687; observed, 535.2692.

## Refinement   

Crystal data, data collection and structure refinement details for (I) and (II) are summarized in Table 3 ▸. Structural models consisting of the target mol­ecules were developed for (I) and (II). Several disordered sites on each mol­ecule were modeled with disorder. In each case, like distances were restrained to be similar. Since the major and minor components of each disordered site are in such close proximity to each other, the displacement parameters were constrained to be equal. Methyl H atom positions, *R*—CH_3_, were optimized by rotation about *R*—C bonds with idealized C—H, *R*–H and H⋯H distances. All hy­droxy and amine H atoms were located in a difference Fourier map in good hydrogen-bonding environments (Hamilton & Ibers, 1968 ▸) and their distances were allowed to refine. The O4—H4*B* distance in (II) was restrained to be 0.84 (2) Å. The remaining H atoms were included as riding idealized contributors. Methyl, hy­droxy and amine H atom*U* values were assigned as 1.5 times *U*_eq_ of the carrier atom; remaining H atom *U* values were assigned as 1.2 times the carrier atom *U*_eq_. On the basis of 2237 unmerged Friedel opposites, the fractional contribution of the inverted twin component was negligible (Flack, 1983 ▸; Flack & Bernardinelli, 2000 ▸) for (I). The absolute structure parameter *y* was calculated using *PLATON* (Spek, 2009 ▸). The resulting value was *y* = −0.010 (19), indicating that the absolute structure has been determined correctly (Hooft *et al.* 2008 ▸). On the basis of 2720 unmerged Friedel opposites, the fractional contribution of the inverted twin component was negligible (Flack, 1983 ▸; Flack & Bernardinelli, 2000 ▸) for (II). The absolute structure parameter *y* was calculated using *PLATON* (Spek, 2009 ▸). The resulting value was *y* = 0.03 (2) indicating that the absolute structure has been determined correctly (Hooft *et al.* 2008 ▸).

## Supplementary Material

Crystal structure: contains datablock(s) I, II. DOI: 10.1107/S2056989015020046/pk2566sup1.cif

Structure factors: contains datablock(s) I. DOI: 10.1107/S2056989015020046/pk2566Isup2.hkl

Supporting information file. DOI: 10.1107/S2056989015020046/pk2566Isup4.cdx

Structure factors: contains datablock(s) II. DOI: 10.1107/S2056989015020046/pk2566IIsup3.hkl

Supporting information file. DOI: 10.1107/S2056989015020046/pk2566IIsup5.cdx

CCDC references: 1432733, 1432732

Additional supporting information:  crystallographic information; 3D view; checkCIF report

## Figures and Tables

**Table 1:** Hydrogen-bond geometry (Å, °) for (I)

*D*—H⋯*A*	*D*—H	H⋯*A*	*D*⋯*A*	*D*—H⋯*A*
O4—H4*B*⋯O5^i^	0.87 (4)	1.94 (4)	2.791 (2)	169 (3)
N2—H2⋯O5	0.83 (3)	2.11 (3)	2.928 (3)	169 (3)
N4—H4⋯O3^ii^	0.84 (3)	2.15 (3)	2.964 (2)	161 (3)

Symmetry codes: (i) 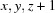; (ii) 
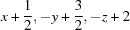
.

**Table 2:** Hydrogen-bond geometry (Å, °) for (II)

*D*—H⋯*A*	*D*—H	H⋯*A*	*D*⋯*A*	*D*—H⋯*A*
N4—H4*C*⋯O4	0.88 (3)	2.60 (3)	3.219 (3)	129 (3)
O4—H4*B*⋯O5^i^	0.82 (1)	1.89 (2)	2.709 (2)	170 (4)

Symmetry code: (i) 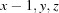.

**Table 3:** Experimental details

	(I)	(II)
Crystal data		
Chemical formula	C_24_H_35_ClN_4_O_5_	C_27_H_39_ClN_4_O_5_
*M*_r_	495.01	535.07
Crystal system, space group	Orthorhombic, *P*2_1_2_1_2	Monoclinic, *P*2_1_
Temperature (K)	193	168
*a*, *b*, *c* (Å)	18.8408 (7), 20.2263 (8), 6.7923 (3)	6.4341 (7), 20.280 (2), 11.0377 (12)
α, β, γ (°)	90, 90, 90	90, 105.248 (1), 90
*V* (Å^3^)	2588.41 (18)	1389.5 (3)
*Z*	4	2
Radiation type	Mo *K*α	Mo *K*α
μ (mm^−1^)	0.19	0.18
Crystal size (mm)	0.37 × 0.36 × 0.29	0.86 × 0.65 × 0.15

Data collection		
Diffractometer	Siemens Platform/APEXII CCD	Siemens Platform/APEXII CCD
Absorption correction	Integration (*SHELXTL*/*XPREP*; Bruker, 2014 ▸)	Integration (*SHELXTL*/*XPREP*; Bruker, 2014 ▸)
*T*_min_, *T*_max_	0.953, 0.960	0.892, 0.980
No. of measured, independent and observed [*I* > 2σ(*I*)] reflections	30342, 5243, 4694	16374, 5627, 5222
*R*_int_	0.027	0.024
(sin θ/λ)_max_ (Å^−1^)	0.623	0.625

Refinement		
*R*[*F* ^2^ > 2σ(*F* ^2^)], *wR*(*F* ^2^), *S*	0.035, 0.085, 1.03	0.032, 0.082, 1.04
No. of reflections	5243	5627
No. of parameters	333	377
No. of restraints	53	14
H-atom treatment	H atoms treated by a mixture of independent and constrained refinement	H atoms treated by a mixture of independent and constrained refinement
Δρ_max_, Δρ_min_ (e Å^−3^)	0.34, −0.43	0.24, −0.21
Absolute structure	Flack (1983 ▸); Hooft *et al.* (2008 ▸); 2720 Friedels	Flack (1983 ▸); Hooft *et al.* (2008 ▸); 2720 Friedels
Absolute structure parameter	−0.008 (18)	0.036 (19)

Computer programs: *APEX2*, *SAINT*, *XPREP* and *XCIF* (Bruker, 2014 ▸), *SHELXS2014* (Sheldrick, 2008 ▸), *SHELXL2014* (Sheldrick, 2015 ▸), *SHELXTL* (Sheldrick, 2008 ▸), *CrystalMaker* (CrystalMaker, 1994 ▸), and *publCIF* (Westrip, 2010 ▸).

## supplementary crystallographic information

### (I) (3S,4aS,8aS)-N-tert-Butyl-2-[(S)-3-(2-chloro-4-nitrobenzamido)-2-hydroxypropyl]decahydroisoquinoline-3-carboxamide Crystal data

**Table:**

C_24_H_35_ClN_4_O_5_	*F*(000) = 1056
*M**_r_* = 495.01	*D*_x_ = 1.270 Mg m^−^^3^
Orthorhombic, *P*2_1_2_1_2	Mo *K*α radiation, λ = 0.71073 Å
Hall symbol: P 2 2ab	Cell parameters from 9966 reflections
*a* = 18.8408 (7) Å	θ = 2.3–24.5°
*b* = 20.2263 (8) Å	µ = 0.19 mm^−^^1^
*c* = 6.7923 (3) Å	*T* = 193 K
*V* = 2588.41 (18) Å^3^	Prism, colourless
*Z* = 4	0.37 × 0.36 × 0.29 mm

### (I) (3S,4aS,8aS)-N-tert-Butyl-2-[(S)-3-(2-chloro-4-nitrobenzamido)-2-hydroxypropyl]decahydroisoquinoline-3-carboxamide Data collection

**Table:**

Siemens Platform/APEXII CCD diffractometer	5243 independent reflections
Radiation source: normal-focus sealed tube	4694 reflections with *I* > 2σ(*I*)
Graphite monochromator	*R*_int_ = 0.027
profile data from φ and ω scans	θ_max_ = 26.3°, θ_min_ = 1.5°
Absorption correction: integration (SHELXTL/XPREP; Bruker, 2014)	*h* = −23→23
*T*_min_ = 0.953, *T*_max_ = 0.960	*k* = −25→25
30342 measured reflections	*l* = −8→8

### (I) (3S,4aS,8aS)-N-tert-Butyl-2-[(S)-3-(2-chloro-4-nitrobenzamido)-2-hydroxypropyl]decahydroisoquinoline-3-carboxamide Refinement

**Table:**

Refinement on *F*^2^	Hydrogen site location: mixed
Least-squares matrix: full	H atoms treated by a mixture of independent and constrained refinement
*R*[*F*^2^ > 2σ(*F*^2^)] = 0.035	*w* = 1/[σ^2^(*F*_o_^2^) + (0.0355*P*)^2^ + 0.5785*P*] where *P* = (*F*_o_^2^ + 2*F*_c_^2^)/3
*wR*(*F*^2^) = 0.085	(Δ/σ)_max_ = 0.001
*S* = 1.03	Δρ_max_ = 0.34 e Å^−^^3^
5243 reflections	Δρ_min_ = −0.43 e Å^−^^3^
333 parameters	Absolute structure: Flack (1983); Hooft *et al.* (2008); 2720 Friedels
53 restraints	Absolute structure parameter: −0.008 (18)

### (I) (3S,4aS,8aS)-N-tert-Butyl-2-[(S)-3-(2-chloro-4-nitrobenzamido)-2-hydroxypropyl]decahydroisoquinoline-3-carboxamide Special details

**Table:**

Experimental. One distinct cell was identified using *APEX2* (Bruker, 2014). Four frame series were integrated and filtered for statistical outliers using *SAINT* (Bruker, 2014) then corrected for absorption by integration using *SHELXTL*/*XPREP* V2005/2 (Bruker, 2014) before using *SAINT*/*SADABS* (Bruker, 2014) to sort, merge, and scale the combined data No decay correction was applied.
Geometry. All e.s.d.'s (except the e.s.d. in the dihedral angle between two l.s. planes) are estimated using the full covariance matrix. The cell e.s.d.'s are taken into account individually in the estimation of e.s.d.'s in distances, angles and torsion angles; correlations between e.s.d.'s in cell parameters are only used when they are defined by crystal symmetry. An approximate (isotropic) treatment of cell e.s.d.'s is used for estimating e.s.d.'s involving l.s. planes.
Refinement. Structure was phased by direct (Sheldrick, 2015). Systematic conditions suggested the ambiguous space group. The space group choice was confirmed by successful convergence of the full-matrix least-squares refinement on *F*^2^. The final map had no significant features. A final analysis of variance between observed and calculated structure factors showed no dependence on amplitude or resolution.

### (I) (3S,4aS,8aS)-N-tert-Butyl-2-[(S)-3-(2-chloro-4-nitrobenzamido)-2-hydroxypropyl]decahydroisoquinoline-3-carboxamide Fractional atomic coordinates and isotropic or equivalent isotropic displacement parameters (Å^2^)

**Table:**

	*x*	*y*	*z*	*U*_iso_*/*U*_eq_	Occ. (<1)
Cl1	0.29551 (15)	0.69804 (7)	0.67519 (15)	0.0735 (5)	0.941 (8)
Cl1B	0.326 (2)	0.6888 (7)	0.664 (3)	0.0735 (5)	0.059 (8)
O1	0.3558 (8)	0.8988 (8)	0.2519 (16)	0.086 (2)	0.60 (2)
O2	0.4046 (8)	0.9713 (5)	0.445 (2)	0.116 (3)	0.60 (2)
N1	0.3808 (6)	0.9169 (5)	0.4064 (12)	0.0715 (17)	0.60 (2)
O1B	0.3725 (13)	0.9134 (12)	0.271 (3)	0.086 (2)	0.40 (2)
O2B	0.4360 (11)	0.9754 (7)	0.453 (3)	0.116 (3)	0.40 (2)
N1B	0.3991 (9)	0.9260 (7)	0.431 (2)	0.0715 (17)	0.40 (2)
C1	0.38040 (11)	0.78173 (14)	0.8790 (4)	0.0477 (6)	
C2	0.34335 (12)	0.77084 (14)	0.7057 (4)	0.0520 (7)	
C3	0.34488 (14)	0.81498 (16)	0.5532 (4)	0.0585 (7)	
H3A	0.3207	0.8062	0.4333	0.070*	
C4	0.38270 (16)	0.87247 (16)	0.5797 (5)	0.0635 (8)	
C5	0.41913 (17)	0.88668 (15)	0.7505 (5)	0.0675 (8)	
H5A	0.4439	0.9272	0.7658	0.081*	
C6	0.41831 (14)	0.84036 (15)	0.8974 (5)	0.0594 (7)	
H6A	0.4443	0.8485	1.0148	0.071*	
C7	0.38161 (12)	0.73281 (14)	1.0445 (4)	0.0478 (6)	
O3	0.32755 (9)	0.71943 (11)	1.1373 (3)	0.0644 (5)	
O4	0.48956 (9)	0.73984 (10)	1.4849 (3)	0.0534 (5)	
H4B	0.5165 (17)	0.7479 (17)	1.585 (5)	0.080*	
O5	0.56221 (7)	0.75831 (7)	0.8387 (2)	0.0372 (3)	
N2	0.44567 (10)	0.70759 (11)	1.0807 (3)	0.0438 (5)	
H2	0.4786 (16)	0.7171 (15)	1.005 (5)	0.066*	
N3	0.60919 (9)	0.66293 (9)	1.1177 (3)	0.0368 (4)	
N4	0.67223 (10)	0.80363 (9)	0.8528 (3)	0.0417 (4)	
H4	0.7145 (16)	0.7950 (14)	0.884 (4)	0.063*	
C8	0.46111 (12)	0.66114 (13)	1.2385 (4)	0.0459 (6)	
H8A	0.4766	0.6186	1.1805	0.055*	
H8B	0.4172	0.6528	1.3145	0.055*	
C9	0.51828 (11)	0.68604 (12)	1.3772 (3)	0.0420 (5)	
H9A	0.5310	0.6499	1.4712	0.050*	
C10	0.58565 (11)	0.70901 (12)	1.2713 (3)	0.0386 (5)	
H10A	0.5767	0.7528	1.2109	0.046*	
H10B	0.6242	0.7144	1.3691	0.046*	
C11	0.65713 (11)	0.69212 (11)	0.9715 (3)	0.0380 (5)	
H11A	0.7036	0.7026	1.0353	0.046*	
C12	0.66899 (13)	0.64166 (11)	0.8056 (4)	0.0410 (5)	
H12A	0.6230	0.6318	0.7413	0.049*	
H12B	0.7010	0.6610	0.7054	0.049*	
C13	0.70138 (14)	0.57736 (12)	0.8829 (4)	0.0494 (6)	
H13A	0.7500	0.5880	0.9326	0.059*	
C14	0.70957 (16)	0.52528 (13)	0.7203 (4)	0.0590 (7)	
H14A	0.7303	0.5467	0.6025	0.071*	
H14B	0.7434	0.4910	0.7656	0.071*	
C15	0.64047 (16)	0.49205 (14)	0.6624 (4)	0.0611 (7)	
H15A	0.6503	0.4565	0.5661	0.073*	
H15B	0.6089	0.5248	0.5988	0.073*	
C16	0.60342 (19)	0.46287 (13)	0.8419 (5)	0.0696 (9)	
H16A	0.6333	0.4276	0.8999	0.084*	
H16B	0.5576	0.4430	0.8019	0.084*	
C17	0.59030 (17)	0.51683 (13)	0.9945 (4)	0.0610 (8)	
H17A	0.5575	0.5502	0.9387	0.073*	
H17B	0.5671	0.4971	1.1115	0.073*	
C18	0.65836 (16)	0.55060 (13)	1.0574 (4)	0.0550 (7)	
H18A	0.6883	0.5165	1.1243	0.066*	
C19	0.64454 (15)	0.60538 (13)	1.2068 (4)	0.0505 (6)	
H19A	0.6145	0.5878	1.3143	0.061*	
H19B	0.6902	0.6196	1.2650	0.061*	
C20	0.62618 (10)	0.75493 (11)	0.8828 (3)	0.0354 (5)	
C21	0.65543 (13)	0.87079 (12)	0.7767 (4)	0.0480 (6)	
C22	0.60827 (17)	0.90585 (14)	0.9239 (5)	0.0679 (8)	
H22A	0.5644	0.8805	0.9422	0.102*	
H22B	0.5967	0.9501	0.8746	0.102*	
H22C	0.6332	0.9097	1.0500	0.102*	
C23	0.62113 (16)	0.86682 (14)	0.5736 (5)	0.0606 (8)	
H23A	0.5739	0.8468	0.5852	0.091*	
H23B	0.6507	0.8397	0.4866	0.091*	
H23C	0.6167	0.9114	0.5184	0.091*	
C24	0.72639 (15)	0.90696 (15)	0.7599 (6)	0.0682 (9)	
H24A	0.7496	0.9079	0.8890	0.102*	
H24B	0.7182	0.9523	0.7145	0.102*	
H24C	0.7569	0.8838	0.6655	0.102*	

### (I) (3S,4aS,8aS)-N-tert-Butyl-2-[(S)-3-(2-chloro-4-nitrobenzamido)-2-hydroxypropyl]decahydroisoquinoline-3-carboxamide Atomic displacement parameters (Å^2^)

**Table:**

	*U*^11^	*U*^22^	*U*^33^	*U*^12^	*U*^13^	*U*^23^
Cl1	0.0607 (12)	0.1010 (6)	0.0588 (5)	−0.0165 (6)	−0.0109 (5)	−0.0190 (5)
Cl1B	0.0607 (12)	0.1010 (6)	0.0588 (5)	−0.0165 (6)	−0.0109 (5)	−0.0190 (5)
O1	0.088 (6)	0.100 (6)	0.068 (2)	0.018 (4)	−0.011 (3)	0.008 (3)
O2	0.141 (8)	0.076 (2)	0.130 (3)	−0.009 (4)	−0.021 (6)	0.021 (2)
N1	0.071 (4)	0.068 (2)	0.075 (3)	0.023 (2)	−0.011 (3)	−0.005 (2)
O1B	0.088 (6)	0.100 (6)	0.068 (2)	0.018 (4)	−0.011 (3)	0.008 (3)
O2B	0.141 (8)	0.076 (2)	0.130 (3)	−0.009 (4)	−0.021 (6)	0.021 (2)
N1B	0.071 (4)	0.068 (2)	0.075 (3)	0.023 (2)	−0.011 (3)	−0.005 (2)
C1	0.0257 (10)	0.0711 (16)	0.0463 (14)	0.0075 (10)	−0.0005 (10)	−0.0111 (13)
C2	0.0330 (12)	0.0761 (18)	0.0471 (14)	0.0110 (12)	−0.0037 (10)	−0.0142 (14)
C3	0.0463 (15)	0.0788 (19)	0.0505 (15)	0.0225 (14)	−0.0057 (12)	−0.0146 (15)
C4	0.0602 (17)	0.0701 (19)	0.0601 (18)	0.0332 (15)	0.0033 (15)	−0.0002 (15)
C5	0.0662 (19)	0.0567 (17)	0.079 (2)	0.0106 (14)	−0.0035 (17)	−0.0068 (16)
C6	0.0464 (14)	0.0732 (19)	0.0586 (17)	0.0057 (14)	−0.0091 (13)	−0.0124 (15)
C7	0.0284 (11)	0.0747 (17)	0.0404 (13)	−0.0032 (11)	−0.0024 (10)	−0.0109 (12)
O3	0.0297 (8)	0.1063 (15)	0.0571 (11)	−0.0049 (9)	0.0044 (8)	0.0005 (12)
O4	0.0413 (9)	0.0750 (13)	0.0440 (9)	0.0114 (9)	−0.0038 (8)	−0.0169 (9)
O5	0.0280 (7)	0.0430 (8)	0.0407 (8)	−0.0008 (6)	−0.0039 (6)	0.0031 (7)
N2	0.0275 (9)	0.0655 (14)	0.0384 (10)	−0.0050 (9)	−0.0011 (8)	−0.0050 (10)
N3	0.0375 (9)	0.0411 (10)	0.0318 (9)	0.0061 (8)	−0.0012 (8)	0.0013 (8)
N4	0.0298 (9)	0.0432 (10)	0.0521 (12)	−0.0032 (8)	−0.0044 (9)	0.0001 (10)
C8	0.0373 (12)	0.0565 (14)	0.0440 (13)	−0.0060 (11)	0.0017 (10)	−0.0014 (12)
C9	0.0383 (12)	0.0525 (13)	0.0352 (11)	0.0052 (10)	−0.0004 (9)	−0.0039 (11)
C10	0.0334 (11)	0.0482 (13)	0.0342 (11)	0.0029 (9)	−0.0056 (9)	−0.0047 (10)
C11	0.0282 (10)	0.0457 (12)	0.0400 (12)	0.0033 (9)	−0.0032 (9)	−0.0016 (10)
C12	0.0393 (12)	0.0460 (12)	0.0378 (12)	0.0059 (10)	0.0050 (10)	0.0019 (10)
C13	0.0482 (13)	0.0531 (14)	0.0470 (14)	0.0192 (12)	0.0032 (12)	0.0006 (12)
C14	0.0673 (17)	0.0557 (16)	0.0540 (16)	0.0224 (14)	0.0116 (14)	0.0021 (13)
C15	0.0818 (19)	0.0466 (14)	0.0549 (16)	0.0096 (13)	0.0141 (15)	−0.0024 (14)
C16	0.097 (2)	0.0436 (14)	0.069 (2)	0.0034 (15)	0.0223 (19)	0.0010 (15)
C17	0.085 (2)	0.0426 (14)	0.0550 (16)	0.0062 (14)	0.0222 (15)	0.0059 (12)
C18	0.0689 (17)	0.0510 (14)	0.0451 (14)	0.0261 (14)	0.0048 (13)	0.0117 (12)
C19	0.0580 (15)	0.0566 (15)	0.0369 (13)	0.0178 (12)	−0.0022 (12)	0.0067 (12)
C20	0.0301 (10)	0.0414 (11)	0.0348 (11)	0.0002 (9)	−0.0006 (9)	−0.0043 (9)
C21	0.0411 (13)	0.0378 (12)	0.0653 (17)	−0.0065 (10)	−0.0019 (12)	−0.0007 (12)
C22	0.0653 (18)	0.0512 (15)	0.087 (2)	0.0017 (14)	0.0045 (17)	−0.0127 (16)
C23	0.0665 (18)	0.0475 (14)	0.0678 (19)	−0.0086 (13)	−0.0126 (15)	0.0125 (14)
C24	0.0540 (16)	0.0529 (16)	0.098 (2)	−0.0193 (13)	−0.0009 (16)	0.0024 (17)

### (I) (3S,4aS,8aS)-N-tert-Butyl-2-[(S)-3-(2-chloro-4-nitrobenzamido)-2-hydroxypropyl]decahydroisoquinoline-3-carboxamide Geometric parameters (Å, º)

**Table:**

Cl1—C2	1.739 (3)	C11—C20	1.522 (3)
Cl1B—C2	1.716 (12)	C11—C12	1.536 (3)
O1—N1	1.207 (7)	C11—H11A	1.0000
O2—N1	1.217 (7)	C12—C13	1.529 (3)
N1—C4	1.482 (7)	C12—H12A	0.9900
O1B—N1B	1.226 (9)	C12—H12B	0.9900
O2B—N1B	1.228 (10)	C13—C14	1.534 (4)
N1B—C4	1.511 (9)	C13—C18	1.535 (4)
C1—C2	1.386 (3)	C13—H13A	1.0000
C1—C6	1.390 (4)	C14—C15	1.517 (4)
C1—C7	1.498 (4)	C14—H14A	0.9900
C2—C3	1.368 (4)	C14—H14B	0.9900
C3—C4	1.376 (5)	C15—C16	1.524 (4)
C3—H3A	0.9500	C15—H15A	0.9900
C4—C5	1.379 (5)	C15—H15B	0.9900
C5—C6	1.368 (4)	C16—C17	1.525 (4)
C5—H5A	0.9500	C16—H16A	0.9900
C6—H6A	0.9500	C16—H16B	0.9900
C7—O3	1.228 (3)	C17—C18	1.514 (4)
C7—N2	1.333 (3)	C17—H17A	0.9900
O4—C9	1.418 (3)	C17—H17B	0.9900
O4—H4B	0.87 (4)	C18—C19	1.525 (4)
O5—C20	1.244 (2)	C18—H18A	1.0000
N2—C8	1.455 (3)	C19—H19A	0.9900
N2—H2	0.83 (3)	C19—H19B	0.9900
N3—C11	1.467 (3)	C21—C22	1.514 (4)
N3—C10	1.467 (3)	C21—C23	1.526 (4)
N3—C19	1.471 (3)	C21—C24	1.528 (3)
N4—C20	1.329 (3)	C22—H22A	0.9800
N4—C21	1.487 (3)	C22—H22B	0.9800
N4—H4	0.84 (3)	C22—H22C	0.9800
C8—C9	1.517 (3)	C23—H23A	0.9800
C8—H8A	0.9900	C23—H23B	0.9800
C8—H8B	0.9900	C23—H23C	0.9800
C9—C10	1.531 (3)	C24—H24A	0.9800
C9—H9A	1.0000	C24—H24B	0.9800
C10—H10A	0.9900	C24—H24C	0.9800
C10—H10B	0.9900		
			
O1—N1—O2	127.3 (9)	H12A—C12—H12B	107.9
O1—N1—C4	121.0 (9)	C12—C13—C14	112.2 (2)
O2—N1—C4	111.6 (9)	C12—C13—C18	110.74 (19)
O1B—N1B—O2B	120.6 (14)	C14—C13—C18	111.5 (2)
O1B—N1B—C4	111.2 (14)	C12—C13—H13A	107.4
O2B—N1B—C4	128.1 (14)	C14—C13—H13A	107.4
C2—C1—C6	118.1 (3)	C18—C13—H13A	107.4
C2—C1—C7	122.7 (2)	C15—C14—C13	113.9 (2)
C6—C1—C7	119.2 (2)	C15—C14—H14A	108.8
C3—C2—C1	121.9 (3)	C13—C14—H14A	108.8
C3—C2—Cl1B	120.7 (7)	C15—C14—H14B	108.8
C1—C2—Cl1B	113.0 (8)	C13—C14—H14B	108.8
C3—C2—Cl1	118.2 (2)	H14A—C14—H14B	107.7
C1—C2—Cl1	119.8 (2)	C14—C15—C16	110.9 (3)
C2—C3—C4	117.6 (3)	C14—C15—H15A	109.5
C2—C3—H3A	121.2	C16—C15—H15A	109.5
C4—C3—H3A	121.2	C14—C15—H15B	109.5
C3—C4—C5	123.0 (3)	C16—C15—H15B	109.5
C3—C4—N1	113.4 (5)	H15A—C15—H15B	108.0
C5—C4—N1	123.7 (5)	C15—C16—C17	109.9 (2)
C3—C4—N1B	128.6 (7)	C15—C16—H16A	109.7
C5—C4—N1B	108.1 (7)	C17—C16—H16A	109.7
C6—C5—C4	117.7 (3)	C15—C16—H16B	109.7
C6—C5—H5A	121.1	C17—C16—H16B	109.7
C4—C5—H5A	121.1	H16A—C16—H16B	108.2
C5—C6—C1	121.6 (3)	C18—C17—C16	112.2 (3)
C5—C6—H6A	119.2	C18—C17—H17A	109.2
C1—C6—H6A	119.2	C16—C17—H17A	109.2
O3—C7—N2	124.9 (3)	C18—C17—H17B	109.2
O3—C7—C1	121.2 (2)	C16—C17—H17B	109.2
N2—C7—C1	113.9 (2)	H17A—C17—H17B	107.9
C9—O4—H4B	109 (2)	C17—C18—C19	111.8 (2)
C7—N2—C8	124.3 (2)	C17—C18—C13	112.8 (2)
C7—N2—H2	118 (2)	C19—C18—C13	110.4 (2)
C8—N2—H2	117 (2)	C17—C18—H18A	107.2
C11—N3—C10	114.32 (17)	C19—C18—H18A	107.2
C11—N3—C19	108.55 (18)	C13—C18—H18A	107.2
C10—N3—C19	110.33 (18)	N3—C19—C18	112.3 (2)
C20—N4—C21	126.27 (19)	N3—C19—H19A	109.2
C20—N4—H4	115 (2)	C18—C19—H19A	109.2
C21—N4—H4	119 (2)	N3—C19—H19B	109.2
N2—C8—C9	112.7 (2)	C18—C19—H19B	109.2
N2—C8—H8A	109.1	H19A—C19—H19B	107.9
C9—C8—H8A	109.1	O5—C20—N4	123.7 (2)
N2—C8—H8B	109.1	O5—C20—C11	120.82 (19)
C9—C8—H8B	109.1	N4—C20—C11	115.44 (18)
H8A—C8—H8B	107.8	N4—C21—C22	108.9 (2)
O4—C9—C8	107.70 (19)	N4—C21—C23	110.9 (2)
O4—C9—C10	109.00 (19)	C22—C21—C23	111.9 (2)
C8—C9—C10	113.42 (19)	N4—C21—C24	106.1 (2)
O4—C9—H9A	108.9	C22—C21—C24	109.8 (2)
C8—C9—H9A	108.9	C23—C21—C24	109.2 (2)
C10—C9—H9A	108.9	C21—C22—H22A	109.5
N3—C10—C9	113.06 (18)	C21—C22—H22B	109.5
N3—C10—H10A	109.0	H22A—C22—H22B	109.5
C9—C10—H10A	109.0	C21—C22—H22C	109.5
N3—C10—H10B	109.0	H22A—C22—H22C	109.5
C9—C10—H10B	109.0	H22B—C22—H22C	109.5
H10A—C10—H10B	107.8	C21—C23—H23A	109.5
N3—C11—C20	111.60 (17)	C21—C23—H23B	109.5
N3—C11—C12	108.59 (18)	H23A—C23—H23B	109.5
C20—C11—C12	108.67 (18)	C21—C23—H23C	109.5
N3—C11—H11A	109.3	H23A—C23—H23C	109.5
C20—C11—H11A	109.3	H23B—C23—H23C	109.5
C12—C11—H11A	109.3	C21—C24—H24A	109.5
C13—C12—C11	111.81 (19)	C21—C24—H24B	109.5
C13—C12—H12A	109.3	H24A—C24—H24B	109.5
C11—C12—H12A	109.3	C21—C24—H24C	109.5
C13—C12—H12B	109.3	H24A—C24—H24C	109.5
C11—C12—H12B	109.3	H24B—C24—H24C	109.5
			
C6—C1—C2—C3	1.8 (4)	C19—N3—C10—C9	−77.0 (2)
C7—C1—C2—C3	−177.6 (2)	O4—C9—C10—N3	−165.33 (18)
C6—C1—C2—Cl1B	158.4 (14)	C8—C9—C10—N3	−45.4 (3)
C7—C1—C2—Cl1B	−20.9 (14)	C10—N3—C11—C20	−52.4 (2)
C6—C1—C2—Cl1	179.7 (2)	C19—N3—C11—C20	−176.07 (18)
C7—C1—C2—Cl1	0.3 (3)	C10—N3—C11—C12	−172.21 (17)
C1—C2—C3—C4	−2.3 (4)	C19—N3—C11—C12	64.2 (2)
Cl1B—C2—C3—C4	−157.2 (15)	N3—C11—C12—C13	−59.4 (2)
Cl1—C2—C3—C4	179.8 (2)	C20—C11—C12—C13	178.98 (19)
C2—C3—C4—C5	0.7 (4)	C11—C12—C13—C14	176.8 (2)
C2—C3—C4—N1	−179.8 (5)	C11—C12—C13—C18	51.5 (3)
C2—C3—C4—N1B	173.4 (9)	C12—C13—C14—C15	−75.8 (3)
O1—N1—C4—C3	−9.0 (13)	C18—C13—C14—C15	49.0 (3)
O2—N1—C4—C3	168.9 (8)	C13—C14—C15—C16	−54.1 (3)
O1—N1—C4—C5	170.5 (10)	C14—C15—C16—C17	57.2 (3)
O2—N1—C4—C5	−11.6 (11)	C15—C16—C17—C18	−57.7 (3)
O1B—N1B—C4—C3	1 (2)	C16—C17—C18—C19	178.8 (2)
O2B—N1B—C4—C3	−175.8 (15)	C16—C17—C18—C13	53.7 (3)
O1B—N1B—C4—C5	174.8 (17)	C12—C13—C18—C17	77.4 (3)
O2B—N1B—C4—C5	−2 (2)	C14—C13—C18—C17	−48.3 (3)
C3—C4—C5—C6	1.4 (4)	C12—C13—C18—C19	−48.5 (3)
N1—C4—C5—C6	−178.1 (6)	C14—C13—C18—C19	−174.2 (2)
N1B—C4—C5—C6	−172.6 (8)	C11—N3—C19—C18	−64.1 (3)
C4—C5—C6—C1	−2.0 (4)	C10—N3—C19—C18	170.0 (2)
C2—C1—C6—C5	0.5 (4)	C17—C18—C19—N3	−70.8 (3)
C7—C1—C6—C5	179.9 (2)	C13—C18—C19—N3	55.7 (3)
C2—C1—C7—O3	−65.7 (3)	C21—N4—C20—O5	4.6 (4)
C6—C1—C7—O3	115.0 (3)	C21—N4—C20—C11	−177.1 (2)
C2—C1—C7—N2	115.6 (3)	N3—C11—C20—O5	−41.3 (3)
C6—C1—C7—N2	−63.7 (3)	C12—C11—C20—O5	78.4 (3)
O3—C7—N2—C8	−1.1 (4)	N3—C11—C20—N4	140.4 (2)
C1—C7—N2—C8	177.5 (2)	C12—C11—C20—N4	−99.9 (2)
C7—N2—C8—C9	−122.8 (3)	C20—N4—C21—C22	64.5 (3)
N2—C8—C9—O4	69.4 (2)	C20—N4—C21—C23	−59.1 (3)
N2—C8—C9—C10	−51.3 (3)	C20—N4—C21—C24	−177.5 (2)
C11—N3—C10—C9	160.33 (18)		

### (I) (3S,4aS,8aS)-N-tert-Butyl-2-[(S)-3-(2-chloro-4-nitrobenzamido)-2-hydroxypropyl]decahydroisoquinoline-3-carboxamide Hydrogen-bond geometry (Å, º)

**Table:**

*D*—H···*A*	*D*—H	H···*A*	*D*···*A*	*D*—H···*A*
O4—H4*B*···O5^i^	0.87 (4)	1.94 (4)	2.791 (2)	169 (3)
N2—H2···O5	0.83 (3)	2.11 (3)	2.928 (3)	169 (3)
N4—H4···O3^ii^	0.84 (3)	2.15 (3)	2.964 (2)	161 (3)

Symmetry codes: (i) *x*, *y*, *z*+1; (ii) *x*+1/2, −*y*+3/2, −*z*+2.

### (II) (3S,4aS,8aS)-N-tert-Butyl-2-{(S)-2-[(S)-1-(2-chloro-4-nitrobenzoyl)pyrrolidin-2-yl]-2-hydroxyethyl}decahydroisoquinoline-3-carboxamide Crystal data

**Table:**

C_27_H_39_ClN_4_O_5_	*F*(000) = 572
*M**_r_* = 535.07	*D*_x_ = 1.279 Mg m^−^^3^
Monoclinic, *P*2_1_	Mo *K*α radiation, λ = 0.71073 Å
Hall symbol: P 2yb	Cell parameters from 7806 reflections
*a* = 6.4341 (7) Å	θ = 2.8–26.3°
*b* = 20.280 (2) Å	µ = 0.18 mm^−^^1^
*c* = 11.0377 (12) Å	*T* = 168 K
β = 105.248 (1)°	Plates, colourless
*V* = 1389.5 (3) Å^3^	0.86 × 0.65 × 0.15 mm
*Z* = 2	

### (II) (3S,4aS,8aS)-N-tert-Butyl-2-{(S)-2-[(S)-1-(2-chloro-4-nitrobenzoyl)pyrrolidin-2-yl]-2-hydroxyethyl}decahydroisoquinoline-3-carboxamide Data collection

**Table:**

Siemens Platform/APEXII CCD diffractometer	5627 independent reflections
Radiation source: normal-focus sealed tube	5222 reflections with *I* > 2σ(*I*)
Graphite monochromator	*R*_int_ = 0.024
profile data from φ and ω scans	θ_max_ = 26.4°, θ_min_ = 1.9°
Absorption correction: integration (SHELXTL/XPREP; Bruker, 2014)	*h* = −8→8
*T*_min_ = 0.892, *T*_max_ = 0.980	*k* = −25→25
16374 measured reflections	*l* = −13→13

### (II) (3S,4aS,8aS)-N-tert-Butyl-2-{(S)-2-[(S)-1-(2-chloro-4-nitrobenzoyl)pyrrolidin-2-yl]-2-hydroxyethyl}decahydroisoquinoline-3-carboxamide Refinement

**Table:**

Refinement on *F*^2^	Hydrogen site location: mixed
Least-squares matrix: full	H atoms treated by a mixture of independent and constrained refinement
*R*[*F*^2^ > 2σ(*F*^2^)] = 0.032	*w* = 1/[σ^2^(*F*_o_^2^) + (0.0447*P*)^2^ + 0.1276*P*] where *P* = (*F*_o_^2^ + 2*F*_c_^2^)/3
*wR*(*F*^2^) = 0.082	(Δ/σ)_max_ < 0.001
*S* = 1.04	Δρ_max_ = 0.24 e Å^−^^3^
5627 reflections	Δρ_min_ = −0.21 e Å^−^^3^
377 parameters	Absolute structure: Flack (1983); Hooft *et al.* (2008); 2720 Friedels
14 restraints	Absolute structure parameter: 0.036 (19)

### (II) (3S,4aS,8aS)-N-tert-Butyl-2-{(S)-2-[(S)-1-(2-chloro-4-nitrobenzoyl)pyrrolidin-2-yl]-2-hydroxyethyl}decahydroisoquinoline-3-carboxamide Special details

**Table:**

Experimental. One distinct cell was identified using *APEX2* (Bruker, 2014). Four frame series were integrated and filtered for statistical outliers using *SAINT* (Bruker, 2014) then corrected for absorption by integration using *SHELXTL*/*XPREP* V2005/2 (Bruker, 2014) before using *SAINT*/*SADABS* (Bruker, 2014) to sort, merge, and scale the combined data. No decay correction was applied.
Geometry. All e.s.d.'s (except the e.s.d. in the dihedral angle between two l.s. planes) are estimated using the full covariance matrix. The cell e.s.d.'s are taken into account individually in the estimation of e.s.d.'s in distances, angles and torsion angles; correlations between e.s.d.'s in cell parameters are only used when they are defined by crystal symmetry. An approximate (isotropic) treatment of cell e.s.d.'s is used for estimating e.s.d.'s involving l.s. planes.
Refinement. Structure was phased by direct (Sheldrick, 2015) methods. Systematic conditions suggested the ambiguous space group. The space group choice was confirmed by successful convergence of the full-matrix least-squares refinement on *F*^2^. The final map had no significant features. A final analysis of variance between observed and calculated structure factors showed little dependence on amplitude and resolution.

### (II) (3S,4aS,8aS)-N-tert-Butyl-2-{(S)-2-[(S)-1-(2-chloro-4-nitrobenzoyl)pyrrolidin-2-yl]-2-hydroxyethyl}decahydroisoquinoline-3-carboxamide Fractional atomic coordinates and isotropic or equivalent isotropic displacement parameters (Å^2^)

**Table:**

	*x*	*y*	*z*	*U*_iso_*/*U*_eq_	Occ. (<1)
Cl1	0.18218 (12)	0.27617 (3)	0.58986 (6)	0.05313 (19)	
O1	0.7185 (5)	0.41190 (18)	0.9393 (3)	0.0657 (8)	0.967 (8)
O2	0.5309 (5)	0.48339 (16)	1.0083 (3)	0.0796 (11)	0.967 (8)
O1B	0.658 (13)	0.391 (2)	0.970 (8)	0.0657 (8)	0.033 (8)
O2B	0.605 (14)	0.4966 (13)	0.961 (9)	0.0796 (11)	0.033 (8)
N1	0.5493 (4)	0.43973 (13)	0.9347 (2)	0.0521 (6)	
O3	−0.3816 (17)	0.3687 (9)	0.5659 (16)	0.064 (2)	0.58 (2)
C7	−0.1974 (15)	0.3696 (13)	0.5438 (6)	0.044 (3)	0.58 (2)
O3B	−0.341 (3)	0.3487 (9)	0.584 (2)	0.064 (2)	0.42 (2)
C7B	−0.209 (2)	0.3832 (18)	0.5437 (8)	0.044 (3)	0.42 (2)
O4	−0.0271 (3)	0.50975 (9)	0.45504 (16)	0.0466 (4)	
H4B	−0.124 (4)	0.5375 (14)	0.441 (3)	0.070*	
O5	0.6776 (3)	0.60681 (10)	0.43924 (17)	0.0497 (5)	
N2	−0.1962 (3)	0.38062 (11)	0.4242 (2)	0.0434 (5)	
N3	0.1541 (3)	0.56188 (9)	0.25105 (17)	0.0349 (4)	
N4	0.3515 (3)	0.61688 (10)	0.48165 (17)	0.0354 (4)	
H4C	0.211 (5)	0.6126 (16)	0.452 (3)	0.053*	
C1	0.1816 (4)	0.34965 (12)	0.6715 (2)	0.0407 (6)	
C2	0.3653 (5)	0.36560 (13)	0.7648 (2)	0.0419 (5)	
H2A	0.4918	0.3393	0.7794	0.050*	
C3	0.3571 (4)	0.42136 (13)	0.8359 (2)	0.0425 (6)	
C4	0.1757 (5)	0.46054 (15)	0.8176 (3)	0.0515 (7)	
H4A	0.1728	0.4977	0.8697	0.062*	
C5	−0.0004 (5)	0.44390 (15)	0.7217 (3)	0.0505 (6)	
H5A	−0.1264	0.4703	0.7071	0.061*	
C6	0.0008 (4)	0.38941 (13)	0.6450 (2)	0.0417 (6)	
C8	−0.0147 (4)	0.40027 (12)	0.3737 (2)	0.0355 (5)	
H8A	0.1254	0.3883	0.4342	0.043*	
C9	−0.0526 (4)	0.35761 (14)	0.2539 (3)	0.0481 (6)	
H9A	−0.0348	0.3849	0.1829	0.058*	
H9B	0.0544	0.3214	0.2674	0.058*	
C10	−0.2747 (10)	0.3296 (4)	0.2237 (6)	0.0504 (15)	0.669 (16)
H10A	−0.2719	0.2816	0.2401	0.060*	0.669 (16)
H10B	−0.3527	0.3379	0.1350	0.060*	0.669 (16)
C11	−0.3755 (11)	0.3670 (4)	0.3125 (7)	0.0423 (15)	0.669 (16)
H11A	−0.4415	0.4086	0.2737	0.051*	0.669 (16)
H11B	−0.4878	0.3400	0.3353	0.051*	0.669 (16)
C10B	−0.2980 (17)	0.3525 (8)	0.2257 (15)	0.0504 (15)	0.331 (16)
H10C	−0.3618	0.3898	0.1705	0.060*	0.331 (16)
H10D	−0.3439	0.3114	0.1777	0.060*	0.331 (16)
C11B	−0.392 (2)	0.3527 (9)	0.3383 (12)	0.0423 (15)	0.331 (16)
H11C	−0.5191	0.3818	0.3268	0.051*	0.331 (16)
H11D	−0.4263	0.3079	0.3630	0.051*	0.331 (16)
C12	−0.0208 (4)	0.47423 (12)	0.3446 (2)	0.0370 (5)	
H12A	−0.1522	0.4846	0.2757	0.044*	
C13	0.1800 (4)	0.49527 (12)	0.3056 (2)	0.0380 (5)	
H13A	0.3061	0.4945	0.3799	0.046*	
H13B	0.2073	0.4636	0.2433	0.046*	
C14	0.3607 (3)	0.59631 (12)	0.2673 (2)	0.0357 (5)	
H14A	0.4542	0.5706	0.2250	0.043*	
C15	0.3232 (4)	0.66580 (12)	0.2091 (2)	0.0369 (5)	
H15A	0.2424	0.6925	0.2564	0.044*	
H15B	0.4642	0.6874	0.2172	0.044*	
C16	0.1982 (4)	0.66434 (13)	0.0704 (2)	0.0380 (5)	
H16A	0.2901	0.6415	0.0230	0.046*	
C17	0.1487 (4)	0.73367 (15)	0.0150 (2)	0.0462 (6)	
H17A	0.2833	0.7598	0.0349	0.055*	
H17B	0.0968	0.7302	−0.0776	0.055*	
C18	−0.0196 (4)	0.76982 (15)	0.0644 (2)	0.0470 (6)	
H18A	0.0388	0.7786	0.1552	0.056*	
H18B	−0.0528	0.8127	0.0209	0.056*	
C19	−0.2244 (4)	0.72936 (14)	0.0435 (2)	0.0451 (6)	
H19A	−0.2910	0.7249	−0.0478	0.054*	
H19B	−0.3279	0.7527	0.0807	0.054*	
C20	−0.1805 (4)	0.66089 (12)	0.1020 (2)	0.0381 (5)	
H20A	−0.3159	0.6351	0.0816	0.046*	
H20B	−0.1306	0.6652	0.1945	0.046*	
C21	−0.0090 (4)	0.62377 (13)	0.0539 (2)	0.0374 (5)	
H21A	−0.0699	0.6159	−0.0380	0.045*	
C22	0.0428 (4)	0.55715 (13)	0.1167 (2)	0.0396 (5)	
H22A	−0.0929	0.5322	0.1070	0.048*	
H22B	0.1344	0.5321	0.0736	0.048*	
C23	0.4782 (3)	0.60558 (11)	0.4055 (2)	0.0359 (5)	
C24	0.4216 (4)	0.62935 (12)	0.6177 (2)	0.0390 (5)	
C25	0.2182 (7)	0.6384 (4)	0.6614 (5)	0.0498 (13)	0.811 (17)
H25A	0.1300	0.5984	0.6430	0.075*	0.811 (17)
H25B	0.1361	0.6760	0.6173	0.075*	0.811 (17)
H25C	0.2573	0.6467	0.7521	0.075*	0.811 (17)
C26	0.5534 (13)	0.5704 (3)	0.6839 (4)	0.0572 (14)	0.811 (17)
H26A	0.4678	0.5300	0.6631	0.086*	0.811 (17)
H26B	0.5913	0.5773	0.7750	0.086*	0.811 (17)
H26C	0.6853	0.5662	0.6559	0.086*	0.811 (17)
C27	0.5599 (11)	0.6921 (3)	0.6388 (7)	0.0569 (15)	0.811 (17)
H27A	0.4787	0.7283	0.5894	0.085*	0.811 (17)
H27B	0.6918	0.6842	0.6124	0.085*	0.811 (17)
H27C	0.5977	0.7037	0.7281	0.085*	0.811 (17)
C25B	0.227 (4)	0.6608 (14)	0.651 (3)	0.0498 (13)	0.189 (17)
H25D	0.0945	0.6390	0.6040	0.075*	0.189 (17)
H25E	0.2201	0.7078	0.6299	0.075*	0.189 (17)
H25F	0.2418	0.6558	0.7415	0.075*	0.189 (17)
C26B	0.461 (5)	0.5602 (8)	0.678 (2)	0.0572 (14)	0.189 (17)
H26D	0.3258	0.5351	0.6569	0.086*	0.189 (17)
H26E	0.5122	0.5647	0.7697	0.086*	0.189 (17)
H26F	0.5692	0.5369	0.6465	0.086*	0.189 (17)
C27B	0.612 (4)	0.6761 (14)	0.659 (3)	0.0569 (15)	0.189 (17)
H27D	0.7446	0.6534	0.6543	0.085*	0.189 (17)
H27E	0.6262	0.6903	0.7457	0.085*	0.189 (17)
H27F	0.5895	0.7148	0.6037	0.085*	0.189 (17)

### (II) (3S,4aS,8aS)-N-tert-Butyl-2-{(S)-2-[(S)-1-(2-chloro-4-nitrobenzoyl)pyrrolidin-2-yl]-2-hydroxyethyl}decahydroisoquinoline-3-carboxamide Atomic displacement parameters (Å^2^)

**Table:**

	*U*^11^	*U*^22^	*U*^33^	*U*^12^	*U*^13^	*U*^23^
Cl1	0.0715 (4)	0.0369 (3)	0.0546 (4)	−0.0005 (3)	0.0230 (3)	−0.0055 (3)
O1	0.0580 (14)	0.0857 (18)	0.0498 (14)	0.0073 (14)	0.0076 (10)	−0.0005 (13)
O2	0.0664 (16)	0.097 (2)	0.0719 (18)	−0.0101 (14)	0.0123 (14)	−0.0422 (16)
O1B	0.0580 (14)	0.0857 (18)	0.0498 (14)	0.0073 (14)	0.0076 (10)	−0.0005 (13)
O2B	0.0664 (16)	0.097 (2)	0.0719 (18)	−0.0101 (14)	0.0123 (14)	−0.0422 (16)
N1	0.0564 (14)	0.0602 (15)	0.0405 (12)	−0.0036 (12)	0.0143 (10)	0.0005 (11)
O3	0.041 (4)	0.092 (8)	0.068 (4)	−0.008 (3)	0.029 (4)	−0.011 (4)
C7	0.0435 (16)	0.040 (10)	0.0557 (16)	−0.004 (3)	0.0269 (13)	−0.0166 (16)
O3B	0.041 (4)	0.092 (8)	0.068 (4)	−0.008 (3)	0.029 (4)	−0.011 (4)
C7B	0.0435 (16)	0.040 (10)	0.0557 (16)	−0.004 (3)	0.0269 (13)	−0.0166 (16)
O4	0.0528 (11)	0.0454 (10)	0.0399 (10)	0.0172 (8)	0.0090 (8)	−0.0097 (8)
O5	0.0298 (8)	0.0613 (12)	0.0524 (11)	0.0106 (8)	0.0011 (7)	−0.0171 (9)
N2	0.0344 (11)	0.0495 (13)	0.0480 (12)	−0.0039 (9)	0.0136 (9)	−0.0135 (10)
N3	0.0322 (10)	0.0374 (11)	0.0333 (10)	0.0008 (8)	0.0054 (8)	−0.0082 (8)
N4	0.0306 (9)	0.0423 (11)	0.0300 (10)	0.0024 (8)	0.0022 (7)	−0.0053 (8)
C1	0.0582 (15)	0.0342 (12)	0.0379 (13)	−0.0028 (11)	0.0273 (12)	−0.0015 (10)
C2	0.0528 (14)	0.0417 (13)	0.0363 (12)	0.0041 (11)	0.0208 (11)	0.0064 (10)
C3	0.0525 (14)	0.0448 (14)	0.0336 (12)	−0.0042 (11)	0.0174 (10)	0.0019 (10)
C4	0.0611 (17)	0.0552 (16)	0.0424 (15)	0.0031 (13)	0.0213 (13)	−0.0126 (12)
C5	0.0487 (15)	0.0599 (17)	0.0469 (15)	0.0071 (13)	0.0196 (12)	−0.0097 (13)
C6	0.0450 (14)	0.0477 (14)	0.0394 (13)	−0.0040 (11)	0.0233 (11)	−0.0054 (11)
C8	0.0330 (11)	0.0394 (12)	0.0350 (12)	0.0029 (9)	0.0104 (9)	−0.0048 (10)
C9	0.0539 (15)	0.0443 (14)	0.0461 (15)	0.0056 (12)	0.0131 (12)	−0.0130 (12)
C10	0.073 (2)	0.037 (4)	0.0420 (16)	−0.021 (3)	0.0166 (17)	−0.018 (3)
C11	0.0325 (16)	0.035 (3)	0.058 (3)	−0.0071 (18)	0.0089 (19)	−0.004 (2)
C10B	0.073 (2)	0.037 (4)	0.0420 (16)	−0.021 (3)	0.0166 (17)	−0.018 (3)
C11B	0.0325 (16)	0.035 (3)	0.058 (3)	−0.0071 (18)	0.0089 (19)	−0.004 (2)
C12	0.0375 (12)	0.0394 (12)	0.0330 (11)	0.0083 (10)	0.0072 (9)	−0.0068 (9)
C13	0.0360 (12)	0.0345 (12)	0.0410 (12)	0.0050 (9)	0.0059 (9)	−0.0089 (10)
C14	0.0265 (10)	0.0438 (13)	0.0364 (12)	0.0029 (9)	0.0077 (9)	−0.0106 (9)
C15	0.0291 (11)	0.0472 (14)	0.0357 (12)	−0.0043 (10)	0.0111 (9)	−0.0062 (10)
C16	0.0304 (11)	0.0535 (15)	0.0325 (12)	−0.0009 (10)	0.0128 (9)	−0.0049 (10)
C17	0.0409 (13)	0.0614 (16)	0.0383 (13)	−0.0066 (12)	0.0139 (10)	0.0040 (12)
C18	0.0487 (14)	0.0487 (14)	0.0450 (14)	−0.0002 (12)	0.0148 (11)	0.0057 (12)
C19	0.0378 (13)	0.0583 (16)	0.0398 (13)	0.0056 (12)	0.0111 (10)	0.0010 (12)
C20	0.0272 (10)	0.0520 (14)	0.0355 (12)	−0.0009 (10)	0.0089 (9)	−0.0009 (10)
C21	0.0322 (11)	0.0523 (14)	0.0267 (11)	−0.0045 (10)	0.0061 (8)	−0.0095 (10)
C22	0.0365 (12)	0.0462 (14)	0.0346 (12)	−0.0017 (10)	0.0066 (9)	−0.0137 (10)
C23	0.0315 (11)	0.0353 (12)	0.0387 (12)	0.0057 (9)	0.0052 (9)	−0.0079 (10)
C24	0.0422 (13)	0.0420 (13)	0.0295 (11)	0.0017 (10)	0.0037 (9)	−0.0019 (9)
C25	0.0545 (17)	0.062 (4)	0.0341 (18)	−0.001 (2)	0.0141 (12)	−0.008 (3)
C26	0.054 (3)	0.066 (2)	0.0469 (18)	0.007 (2)	0.005 (2)	0.0227 (17)
C27	0.072 (3)	0.058 (3)	0.039 (3)	−0.015 (3)	0.012 (2)	−0.015 (2)
C25B	0.0545 (17)	0.062 (4)	0.0341 (18)	−0.001 (2)	0.0141 (12)	−0.008 (3)
C26B	0.054 (3)	0.066 (2)	0.0469 (18)	0.007 (2)	0.005 (2)	0.0227 (17)
C27B	0.072 (3)	0.058 (3)	0.039 (3)	−0.015 (3)	0.012 (2)	−0.015 (2)

### (II) (3S,4aS,8aS)-N-tert-Butyl-2-{(S)-2-[(S)-1-(2-chloro-4-nitrobenzoyl)pyrrolidin-2-yl]-2-hydroxyethyl}decahydroisoquinoline-3-carboxamide Geometric parameters (Å, º)

**Table:**

Cl1—C1	1.742 (2)	C12—H12A	1.0000
O1—N1	1.215 (3)	C13—H13A	0.9900
O2—N1	1.228 (3)	C13—H13B	0.9900
O1B—N1	1.210 (15)	C14—C23	1.525 (3)
O2B—N1	1.219 (15)	C14—C15	1.541 (3)
N1—C3	1.466 (3)	C14—H14A	1.0000
O3—C7B	1.236 (18)	C15—C16	1.531 (3)
O3—C7	1.271 (11)	C15—H15A	0.9900
C7—O3B	1.20 (2)	C15—H15B	0.9900
C7—N2	1.341 (7)	C16—C17	1.533 (4)
C7—C6	1.512 (7)	C16—C21	1.536 (3)
O3B—C7B	1.271 (14)	C16—H16A	1.0000
C7B—N2	1.343 (10)	C17—C18	1.522 (4)
C7B—C6	1.513 (10)	C17—H17A	0.9900
O4—C12	1.425 (3)	C17—H17B	0.9900
O4—H4B	0.824 (14)	C18—C19	1.518 (4)
O5—C23	1.239 (3)	C18—H18A	0.9900
N2—C8	1.475 (3)	C18—H18B	0.9900
N2—C11	1.476 (5)	C19—C20	1.526 (4)
N2—C11B	1.477 (10)	C19—H19A	0.9900
N3—C22	1.470 (3)	C19—H19B	0.9900
N3—C14	1.470 (3)	C20—C21	1.541 (3)
N3—C13	1.471 (3)	C20—H20A	0.9900
N4—C23	1.336 (3)	C20—H20B	0.9900
N4—C24	1.472 (3)	C21—C22	1.515 (4)
N4—H4C	0.88 (3)	C21—H21A	1.0000
C1—C6	1.382 (4)	C22—H22A	0.9900
C1—C2	1.387 (4)	C22—H22B	0.9900
C2—C3	1.385 (4)	C24—C25	1.520 (4)
C2—H2A	0.9500	C24—C27B	1.524 (13)
C3—C4	1.382 (4)	C24—C27	1.535 (5)
C4—C5	1.374 (4)	C24—C26	1.535 (4)
C4—H4A	0.9500	C24—C25B	1.536 (13)
C5—C6	1.393 (4)	C24—C26B	1.545 (12)
C5—H5A	0.9500	C25—H25A	0.9800
C8—C12	1.532 (3)	C25—H25B	0.9800
C8—C9	1.545 (3)	C25—H25C	0.9800
C8—H8A	1.0000	C26—H26A	0.9800
C9—C10	1.492 (6)	C26—H26B	0.9800
C9—C10B	1.531 (11)	C26—H26C	0.9800
C9—H9A	0.9900	C27—H27A	0.9800
C9—H9B	0.9900	C27—H27B	0.9800
C10—C11	1.514 (7)	C27—H27C	0.9800
C10—H10A	0.9900	C25B—H25D	0.9800
C10—H10B	0.9900	C25B—H25E	0.9800
C11—H11A	0.9900	C25B—H25F	0.9800
C11—H11B	0.9900	C26B—H26D	0.9800
C10B—C11B	1.519 (11)	C26B—H26E	0.9800
C10B—H10C	0.9900	C26B—H26F	0.9800
C10B—H10D	0.9900	C27B—H27D	0.9800
C11B—H11C	0.9900	C27B—H27E	0.9800
C11B—H11D	0.9900	C27B—H27F	0.9800
C12—C13	1.526 (3)		
			
O1B—N1—O2B	126 (3)	N3—C14—C23	112.03 (19)
O1—N1—O2	123.3 (3)	N3—C14—C15	110.24 (17)
O1B—N1—C3	110 (3)	C23—C14—C15	106.64 (18)
O1—N1—C3	119.0 (2)	N3—C14—H14A	109.3
O2B—N1—C3	124 (3)	C23—C14—H14A	109.3
O2—N1—C3	117.8 (3)	C15—C14—H14A	109.3
C7B—O3—C7	13 (3)	C16—C15—C14	112.51 (19)
O3B—C7—O3	22.9 (9)	C16—C15—H15A	109.1
O3B—C7—N2	128.8 (12)	C14—C15—H15A	109.1
O3—C7—N2	116.0 (12)	C16—C15—H15B	109.1
O3B—C7—C6	113.6 (12)	C14—C15—H15B	109.1
O3—C7—C6	120.7 (10)	H15A—C15—H15B	107.8
N2—C7—C6	117.5 (7)	C15—C16—C17	112.4 (2)
C7—O3B—C7B	13 (3)	C15—C16—C21	109.89 (19)
O3—C7B—O3B	22.8 (9)	C17—C16—C21	111.2 (2)
O3—C7B—N2	118.4 (11)	C15—C16—H16A	107.7
O3B—C7B—N2	122.9 (19)	C17—C16—H16A	107.7
O3—C7B—C6	123.1 (11)	C21—C16—H16A	107.7
O3B—C7B—C6	109.5 (15)	C18—C17—C16	113.0 (2)
N2—C7B—C6	117.2 (9)	C18—C17—H17A	109.0
C12—O4—H4B	112 (2)	C16—C17—H17A	109.0
C7—N2—C7B	12 (3)	C18—C17—H17B	109.0
C7—N2—C8	128.8 (4)	C16—C17—H17B	109.0
C7B—N2—C8	128.2 (5)	H17A—C17—H17B	107.8
C7—N2—C11	125.8 (5)	C19—C18—C17	110.9 (2)
C8—N2—C11	105.0 (4)	C19—C18—H18A	109.5
C7—N2—C11B	110.1 (7)	C17—C18—H18A	109.5
C7B—N2—C11B	112.6 (8)	C19—C18—H18B	109.5
C8—N2—C11B	119.2 (6)	C17—C18—H18B	109.5
C22—N3—C14	109.84 (18)	H18A—C18—H18B	108.1
C22—N3—C13	109.01 (18)	C18—C19—C20	111.8 (2)
C14—N3—C13	112.62 (18)	C18—C19—H19A	109.3
C23—N4—C24	126.7 (2)	C20—C19—H19A	109.3
C23—N4—H4C	118.8 (19)	C18—C19—H19B	109.3
C24—N4—H4C	114.2 (19)	C20—C19—H19B	109.3
C6—C1—C2	121.8 (2)	H19A—C19—H19B	107.9
C6—C1—Cl1	120.4 (2)	C19—C20—C21	111.6 (2)
C2—C1—Cl1	117.8 (2)	C19—C20—H20A	109.3
C3—C2—C1	117.3 (2)	C21—C20—H20A	109.3
C3—C2—H2A	121.3	C19—C20—H20B	109.3
C1—C2—H2A	121.3	C21—C20—H20B	109.3
C4—C3—C2	122.9 (2)	H20A—C20—H20B	108.0
C4—C3—N1	118.5 (2)	C22—C21—C16	110.13 (19)
C2—C3—N1	118.6 (2)	C22—C21—C20	111.7 (2)
C5—C4—C3	117.7 (2)	C16—C21—C20	112.0 (2)
C5—C4—H4A	121.2	C22—C21—H21A	107.6
C3—C4—H4A	121.2	C16—C21—H21A	107.6
C4—C5—C6	121.9 (3)	C20—C21—H21A	107.6
C4—C5—H5A	119.1	N3—C22—C21	113.16 (18)
C6—C5—H5A	119.1	N3—C22—H22A	108.9
C1—C6—C5	118.2 (2)	C21—C22—H22A	108.9
C1—C6—C7	120.0 (10)	N3—C22—H22B	108.9
C5—C6—C7	121.4 (9)	C21—C22—H22B	108.9
C1—C6—C7B	130.1 (13)	H22A—C22—H22B	107.8
C5—C6—C7B	111.7 (13)	O5—C23—N4	124.3 (2)
C7—C6—C7B	11 (2)	O5—C23—C14	120.3 (2)
N2—C8—C12	111.36 (19)	N4—C23—C14	115.23 (18)
N2—C8—C9	102.10 (19)	N4—C24—C25	106.6 (3)
C12—C8—C9	112.3 (2)	N4—C24—C27B	114.8 (14)
N2—C8—H8A	110.3	N4—C24—C27	107.9 (3)
C12—C8—H8A	110.3	C25—C24—C27	111.4 (4)
C9—C8—H8A	110.3	N4—C24—C26	109.6 (3)
C10—C9—C8	109.3 (3)	C25—C24—C26	110.9 (3)
C10B—C9—C8	97.7 (6)	C27—C24—C26	110.3 (3)
C10—C9—H9A	109.8	N4—C24—C25B	105.5 (12)
C8—C9—H9A	109.8	C27B—C24—C25B	108.3 (18)
C10—C9—H9B	109.8	N4—C24—C26B	104.9 (9)
C8—C9—H9B	109.8	C27B—C24—C26B	114.1 (13)
H9A—C9—H9B	108.3	C25B—C24—C26B	108.9 (13)
C9—C10—C11	102.3 (6)	C24—C25—H25A	109.5
C9—C10—H10A	111.3	C24—C25—H25B	109.5
C11—C10—H10A	111.3	H25A—C25—H25B	109.5
C9—C10—H10B	111.3	C24—C25—H25C	109.5
C11—C10—H10B	111.3	H25A—C25—H25C	109.5
H10A—C10—H10B	109.2	H25B—C25—H25C	109.5
N2—C11—C10	105.1 (5)	C24—C26—H26A	109.5
N2—C11—H11A	110.7	C24—C26—H26B	109.5
C10—C11—H11A	110.7	H26A—C26—H26B	109.5
N2—C11—H11B	110.7	C24—C26—H26C	109.5
C10—C11—H11B	110.7	H26A—C26—H26C	109.5
H11A—C11—H11B	108.8	H26B—C26—H26C	109.5
C11B—C10B—C9	116.4 (11)	C24—C27—H27A	109.5
C11B—C10B—H10C	108.2	C24—C27—H27B	109.5
C9—C10B—H10C	108.2	H27A—C27—H27B	109.5
C11B—C10B—H10D	108.2	C24—C27—H27C	109.5
C9—C10B—H10D	108.2	H27A—C27—H27C	109.5
H10C—C10B—H10D	107.3	H27B—C27—H27C	109.5
N2—C11B—C10B	94.0 (9)	C24—C25B—H25D	109.5
N2—C11B—H11C	112.9	C24—C25B—H25E	109.5
C10B—C11B—H11C	112.9	H25D—C25B—H25E	109.5
N2—C11B—H11D	112.9	C24—C25B—H25F	109.5
C10B—C11B—H11D	112.9	H25D—C25B—H25F	109.5
H11C—C11B—H11D	110.3	H25E—C25B—H25F	109.5
O4—C12—C13	108.45 (19)	C24—C26B—H26D	109.5
O4—C12—C8	108.7 (2)	C24—C26B—H26E	109.5
C13—C12—C8	110.82 (18)	H26D—C26B—H26E	109.5
O4—C12—H12A	109.6	C24—C26B—H26F	109.5
C13—C12—H12A	109.6	H26D—C26B—H26F	109.5
C8—C12—H12A	109.6	H26E—C26B—H26F	109.5
N3—C13—C12	111.04 (18)	C24—C27B—H27D	109.5
N3—C13—H13A	109.4	C24—C27B—H27E	109.5
C12—C13—H13A	109.4	H27D—C27B—H27E	109.5
N3—C13—H13B	109.4	C24—C27B—H27F	109.5
C12—C13—H13B	109.4	H27D—C27B—H27F	109.5
H13A—C13—H13B	108.0	H27E—C27B—H27F	109.5
			
C7B—O3—C7—O3B	154 (6)	O3B—C7B—C6—C5	−89 (3)
C7B—O3—C7—N2	−77 (3)	N2—C7B—C6—C5	125 (2)
C7B—O3—C7—C6	76 (3)	O3—C7B—C6—C7	87 (5)
O3—C7—O3B—C7B	−26 (5)	O3B—C7B—C6—C7	67 (4)
N2—C7—O3B—C7B	−88 (4)	N2—C7B—C6—C7	−80 (4)
C6—C7—O3B—C7B	88 (3)	C7—N2—C8—C12	−100.6 (15)
C7—O3—C7B—O3B	−25 (5)	C7B—N2—C8—C12	−85 (2)
C7—O3—C7B—N2	83 (4)	C11—N2—C8—C12	86.4 (4)
C7—O3—C7B—C6	−84 (4)	C11B—N2—C8—C12	96.6 (9)
C7—O3B—C7B—O3	153 (6)	C7—N2—C8—C9	139.3 (15)
C7—O3B—C7B—N2	68 (4)	C7B—N2—C8—C9	155 (2)
C7—O3B—C7B—C6	−76 (3)	C11—N2—C8—C9	−33.6 (4)
O3B—C7—N2—C7B	95 (4)	C11B—N2—C8—C9	−23.5 (9)
O3—C7—N2—C7B	72 (4)	N2—C8—C9—C10	14.1 (5)
C6—C7—N2—C7B	−81 (4)	C12—C8—C9—C10	−105.3 (4)
O3B—C7—N2—C8	−174 (2)	N2—C8—C9—C10B	29.1 (7)
O3—C7—N2—C8	163.8 (14)	C12—C8—C9—C10B	−90.3 (7)
C6—C7—N2—C8	11 (3)	C8—C9—C10—C11	10.2 (8)
O3—C7—N2—C11	−25 (3)	C7—N2—C11—C10	−131.3 (15)
C6—C7—N2—C11	−177.9 (10)	C8—N2—C11—C10	42.0 (7)
O3B—C7—N2—C11B	−10 (4)	C9—C10—C11—N2	−31.3 (9)
O3—C7—N2—C11B	−32 (3)	C8—C9—C10B—C11B	−31.8 (15)
C6—C7—N2—C11B	174.6 (15)	C7—N2—C11B—C10B	−161.4 (15)
O3—C7B—N2—C7	−87 (5)	C7B—N2—C11B—C10B	−174 (2)
O3B—C7B—N2—C7	−61 (4)	C8—N2—C11B—C10B	4.3 (16)
C6—C7B—N2—C7	80 (4)	C9—C10B—C11B—N2	18.3 (18)
O3—C7B—N2—C8	174.9 (19)	N2—C8—C12—O4	55.0 (2)
O3B—C7B—N2—C8	−159.1 (18)	C9—C8—C12—O4	168.76 (19)
C6—C7B—N2—C8	−18 (4)	N2—C8—C12—C13	174.05 (19)
O3—C7B—N2—C11B	−7 (4)	C9—C8—C12—C13	−72.1 (2)
O3B—C7B—N2—C11B	19 (4)	C22—N3—C13—C12	−85.1 (2)
C6—C7B—N2—C11B	161 (2)	C14—N3—C13—C12	152.70 (18)
C6—C1—C2—C3	3.5 (3)	O4—C12—C13—N3	−73.4 (2)
Cl1—C1—C2—C3	−174.92 (18)	C8—C12—C13—N3	167.38 (18)
C1—C2—C3—C4	0.3 (4)	C22—N3—C14—C23	177.20 (18)
C1—C2—C3—N1	−179.6 (2)	C13—N3—C14—C23	−61.1 (2)
O1B—N1—C3—C4	155 (6)	C22—N3—C14—C15	58.6 (2)
O1—N1—C3—C4	−168.9 (3)	C13—N3—C14—C15	−179.68 (18)
O2B—N1—C3—C4	−36 (7)	N3—C14—C15—C16	−56.0 (2)
O2—N1—C3—C4	10.2 (4)	C23—C14—C15—C16	−177.86 (18)
O1B—N1—C3—C2	−26 (6)	C14—C15—C16—C17	176.37 (19)
O1—N1—C3—C2	10.9 (4)	C14—C15—C16—C21	52.0 (2)
O2B—N1—C3—C2	144 (7)	C15—C16—C17—C18	−70.8 (3)
O2—N1—C3—C2	−169.9 (3)	C21—C16—C17—C18	52.9 (3)
C2—C3—C4—C5	−2.2 (4)	C16—C17—C18—C19	−55.0 (3)
N1—C3—C4—C5	177.7 (2)	C17—C18—C19—C20	55.7 (3)
C3—C4—C5—C6	0.4 (4)	C18—C19—C20—C21	−55.3 (3)
C2—C1—C6—C5	−5.2 (4)	C15—C16—C21—C22	−51.3 (2)
Cl1—C1—C6—C5	173.2 (2)	C17—C16—C21—C22	−176.4 (2)
C2—C1—C6—C7	−179.1 (6)	C15—C16—C21—C20	73.6 (2)
Cl1—C1—C6—C7	−0.7 (6)	C17—C16—C21—C20	−51.5 (3)
C2—C1—C6—C7B	176.1 (9)	C19—C20—C21—C22	177.21 (19)
Cl1—C1—C6—C7B	−5.5 (10)	C19—C20—C21—C16	53.1 (3)
C4—C5—C6—C1	3.2 (4)	C14—N3—C22—C21	−61.1 (2)
C4—C5—C6—C7	177.0 (7)	C13—N3—C22—C21	175.09 (19)
C4—C5—C6—C7B	−177.9 (8)	C16—C21—C22—N3	57.2 (2)
O3B—C7—C6—C1	105 (2)	C20—C21—C22—N3	−67.9 (2)
O3—C7—C6—C1	129 (2)	C24—N4—C23—O5	−3.2 (4)
N2—C7—C6—C1	−79 (2)	C24—N4—C23—C14	−178.2 (2)
O3B—C7—C6—C5	−69 (3)	N3—C14—C23—O5	149.4 (2)
O3—C7—C6—C5	−44 (3)	C15—C14—C23—O5	−89.9 (2)
N2—C7—C6—C5	107.5 (16)	N3—C14—C23—N4	−35.4 (3)
O3B—C7—C6—C7B	−95 (4)	C15—C14—C23—N4	85.3 (2)
O3—C7—C6—C7B	−71 (4)	C23—N4—C24—C25	−179.4 (4)
N2—C7—C6—C7B	81 (4)	C23—N4—C24—C27B	43.0 (15)
O3—C7B—C6—C1	110 (3)	C23—N4—C24—C27	60.9 (4)
O3B—C7B—C6—C1	90 (2)	C23—N4—C24—C26	−59.3 (5)
N2—C7B—C6—C1	−57 (3)	C23—N4—C24—C25B	162.1 (12)
O3—C7B—C6—C5	−69 (3)	C23—N4—C24—C26B	−83.0 (13)

### (II) (3S,4aS,8aS)-N-tert-Butyl-2-{(S)-2-[(S)-1-(2-chloro-4-nitrobenzoyl)pyrrolidin-2-yl]-2-hydroxyethyl}decahydroisoquinoline-3-carboxamide Hydrogen-bond geometry (Å, º)

**Table:**

*D*—H···*A*	*D*—H	H···*A*	*D*···*A*	*D*—H···*A*
N4—H4*C*···O4	0.88 (3)	2.60 (3)	3.219 (3)	129 (3)
O4—H4*B*···O5^i^	0.82 (1)	1.89 (2)	2.709 (2)	170 (4)

Symmetry code: (i) *x*−1, *y*, *z*.
